# Yolk–Shell CoNi@N-Doped Carbon-CoNi@CNTs for Enhanced Microwave Absorption, Photothermal, Anti-Corrosion, and Antimicrobial Properties

**DOI:** 10.1007/s40820-024-01626-8

**Published:** 2025-02-26

**Authors:** Qiqin Liang, Mukun He, Beibei Zhan, Hua Guo, Xiaosi Qi, Yunpeng Qu, Yali Zhang, Wei Zhong, Junwei Gu

**Affiliations:** 1 College of Physics, Guizhou Province Key Laboratory for Photoelectrics Technology and Application, Guizhou University, Guiyang City, 550025 People’s Republic of China; 2 Shaanxi Key Laboratory of Macromolecular Science and Technology, School of Chemistry and Chemical Engineering, Northwestern Polytechnical University, Xi’an, 710072 People’s Republic of China; 3 National Laboratory of Solid State Microstructures and Jiangsu Provincial Laboratory for NanoTechnology, Nanjing University, Nanjing, 210093 People’s Republic of China

**Keywords:** Sea urchin, Like yolk, Shell structure, CoNi@N, Doped carbon, CoNi@carbon nanotubes, Mixed, Dimensional nanocomposites, Microwave absorption, Photothermal and antimicrobial

## Abstract

The previous studies mainly focused on improving microwave absorbing (MA) performances of MA materials. Even so, these designed MA materials were very difficult to be employed in complex and changing environments owing to their single-functionalities. Herein, a combined Prussian blue analogues derived and catalytical chemical vapor deposition strategy was proposed to produce hierarchical cubic sea urchin-like yolk–shell CoNi@N-doped carbon (NC)-CoNi@carbon nanotubes (CNTs) mixed-dimensional multicomponent nanocomposites (MCNCs), which were composed of zero-dimensional CoNi nanoparticles, three-dimensional NC nanocubes and one-dimensional CNTs. Because of good impedance matching and attenuation characteristics, the designed CoNi@NC-CoNi@CNTs mixed-dimensional MCNCs exhibited excellent MA performances, which achieved a minimum reflection loss (*RL*_min_) of −71.70 dB at 2.78 mm and Radar Cross section value of −53.23 dB m^2^. More importantly, the acquired results demonstrated that CoNi@NC-CoNi@CNTs MCNCs presented excellent photothermal, antimicrobial and anti-corrosion properties owing to their hierarchical cubic sea urchin-like yolk–shell structure, highlighting their potential multifunctional applications. It could be seen that this finding not only presented a generalizable route to produce hierarchical cubic sea urchin-like yolk–shell magnetic NC-CNTs-based mixed-dimensional MCNCs, but also provided an effective strategy to develop multifunctional MCNCs and improve their environmental adaptabilities.

**Supplementary Information:**

The online version contains supplementary material available at 10.1007/s40820-024-01626-8.

## Highlights

- Hierarchical cubic sea urchin-like yolk-shell CoNi@N-doped carbon (NC)-CoNi@carbon nanotubes (CNTs) mixed-dimensional MCNCs with different CNTs contents could be availably and selectively synthesized.
- Owing to the generated hierarchical cubic sea urchin like yolk-shell structure, CoNi@NC-CoNi@CNTs display marvelous hydrophobic, corrosion resistance and MA performance.
- The construction of components and special microstructure make CoNi@NC-CoNi@CNTs MCNCs display terrific photothermal and antimicrobial (e g. only 1 mg·mL^-1^ concentration, ~84.03% antimicrobial rate) proper ties.

**Supplementary Information:**

The online version contains supplementary material available at 10.1007/s40820-024-01626-8.

## Graphical abstract

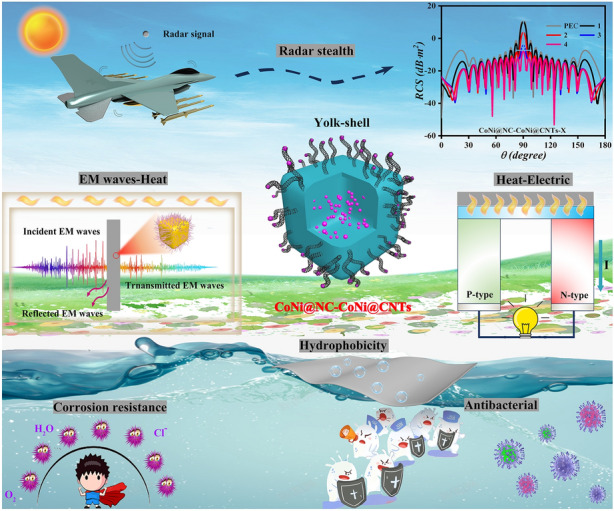

## Introduction

With the rapid development of modern science and electronic technology, microwave absorbing (MA) materials have shown an indispensable role in the fields of wireless communication, electromagnetic (EM) shielding, stealth technology and energy conversion [1–3]. Until now, a variety of methods and strategies have been proposed to improve MA performances including magnetic–dielectric synergy, interface engineering, mixed dimension, etc. [4–6]. To make best of magnetic–dielectric synergistic effect [7], Qiao et al. introduced the magnetic component Ni and dielectric component MnO into the carbon aerogel thus regulating the impedance matching of carbon aerogel. Owing to excellent magnetic–dielectric synergistic effect. The designed Ni/MnO-carbon aerogel can obtain satisfactory MA performance with minimum reflection loss (*RL*_min_) of -64.09 dB and effective absorption bandwidth (EAB) of 7.36 GHz [8]. To build abundant interfaces, Zhang et al. constructed a titanium nitride (TiN) nanotube/polydimethylsiloxane (PDMS) composite. Benefiting from the rich heterogenous interfaces between TiN nanotubes and PDMS, the TiN nanotubes/PDMS composite displayed significantly enhanced MA properties compared with TiN nanotubes [9]. Sun and his co-workers elaborately designed a “one-dimensional (1D) carbon nanotubes (CNTs)/zero-dimensional (0D) Fe_2_N nanoparticle” heterostructure with dual properties. The results showed that the prepared mixed-dimensional encapsulated structure Fe_2_N@CNTs exhibited excellent MA performance due to the multiple loss mechanism and excellent interfacial effects [10]. All in all, the above-mentioned strategies are largely beneficial to improve the MA performances including strong absorption, wide bandwidth and thin thickness [11, 12]. Nevertheless, most MA materials are still difficult to face the complex and changeable natural environment owing to the lack of multifunctionality. Therefore, studies of other properties including hydrophobic, corrosion resistance and antibacterial properties are very important to evaluate their potential applications in the marine environment [13, 14].

It is always known that the physical and chemical properties of materials are determined by their categories and structures. Metal–organic frameworks (MOFs) is a nano-porous structure composed of metal ions (or clusters) and organic ligands, which has the advantages of large specific surface area, rich porosity, structural and componential diversities [15, 16]. More importantly, the previous results revealed that MOFs derivatives could well inherit the advantages of MOFs precursors and metal/carbon components, which presented excellent properties including good thermal conductivity, high biocompatibility, low toxicity and light weight due to the special structures and carbon skeleton [17, 18]. Therefore, constructing MOFs derivatives is a desirable strategy to develop high efficiency MA materials with the features of low density, strong absorption and wide bandwidth. However, a single structure (core–shell, yolk–shell), weak chemical stability, and great functional integration difficulty of MOF derivatives limit their applications in multifunctional materials. It has been reported that the mixed-dimension and hierarchical structure are an effective strategy to solve the above problems: (i) Giving MOF derivatives higher structural flexibility to more easily integrate with other functional materials [19], (ii) structural units of different dimensions often have different physical and chemical properties, allowing the MOFs derivatives to have multiple functional properties simultaneously [20], and (iii) enhance the chemical and thermal stability of MOF derivatives [21]. In addition, CNTs are the preferred candidates for preparing multifunctional materials due to their excellent mechanical properties, electrical conductivity, thermal conductivity, chemical stability and so on [22, 23].

Inspired by the mentioned above and our previous work [24, 25], magnetic carbon-based CNTs multicomponent nanocomposites (MCNCs) with mixed-dimensional hierarchical structure derived from MOFs is a promising way to expand the multifunctionality. Herein, we report a scalable strategy to fabricate hierarchical cubic sea urchin-like yolk–shell CoNi@N-doped carbon (NC)-CoNi@CNTs mixed-dimensional MCNCs, which are composed of 0D CoNi nanoparticles, three-dimensional (3D) NC nanocubes and 1D CNTs through a combined Prussian blue analogues (PBAs) derived and catalytical chemical vapor deposition strategy. Owing to the special structure and synergistic effect among the component substances, the designed CoNi@NC-CoNi@CNTs mixed-dimensional MCNCs present excellent MA performances, photothermal, antimicrobial and anti-corrosion properties, highlighting their potential multifunctional applications.

## Experimental Section

In a typical experiment, yolk–shell CoNi@NC-CoNi@CNTs MCNCs can be selectively and efficiently produced through a facile combined co-precipitation route and catalytic chemical vapor deposition. The adopted experimental routes and measurements are provided in Supporting Information. As summarized in Table S1 (Supporting Information), the growth of CNTs is designed at different temperatures (660, 760, and 860 °C) and times (2 and 4 h) to produce CoNi@NC-CoNi@CNTs MCNCs, which are marked as CoNi@NC-CoNi@CNTs-1, CoNi@NC-CoNi@CNTs-2, CoNi@NC-CoNi@CNTs-3, and CoNi@NC-CoNi@CNTs-4, respectively.

## Results and Discussion

### Design and Fabrication of PBAs-Derived CoNi@NC-CoNi@CNTs MCNCs

The synthesis route of 3D cubic CoNi PBAs derived cubic sea urchin-like yolk–shell CoNi@NC-CoNi@CNTs mixed-dimensional MCNCs is schematically depicted in Fig. S1a. The main production process can be summarized into two steps: Firstly, the dissociated Ni^2+^ and [Co(CN)_6_]^3−^ aggregate under the supersaturation by van der Waals forces into primary nanoparticles, which eventually grow into CoNi PBAs with 3D cubic morphology under the control of trisodium citrate dihydrate through co-precipitation process. Subsequently, the acquired CoNi PBAs and melamine are served as catalyst precursor and carbon source to grow CNTs via a catalytic chemical vapor deposition process, which results in the production of cubic sea urchin-like yolk–shell CoNi@NC-CoNi@CNTs mixed-dimensional MCNCs. The X-ray powder diffractometry (XRD) outcome (Fig. S1b) demonstrates that all the diffraction peaks of catalyst precursor can be assigned to the phase of Ni_3_[Co(CN)_6_]_2_·12H_2_O (JCPDS No. 89–3738) [26], which suggests the generation of CoNi PBAs. In addition, the FESEM investigation (Fig. S1c, d) reveals that the as-prepared CoNi PBAs presents a typical regular cube-like morphology with a smooth surface and relatively uniform size.

**Fig. 1:**
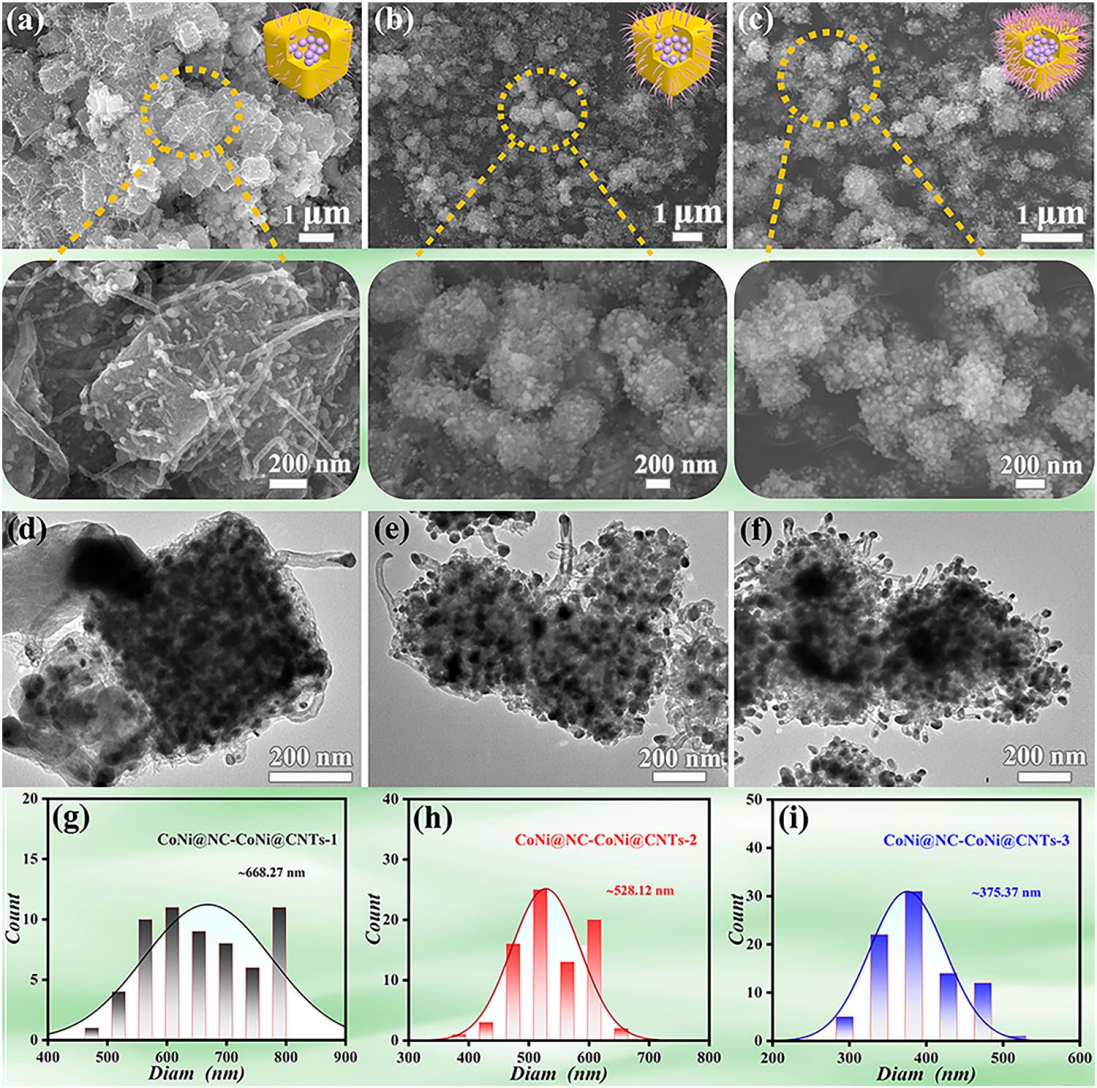
**a-c** FESEM, **d-f** TEM images and **g-i** size distribution chart of CoNi@NC-CoNi@CNTs-1, CoNi@NC-CoNi@CNTs-2, and CoNi@NC-CoNi@CNTs-3

To determine the morphology, composition and chemical valence, the investigations of FESEM, XRD patterns, Raman spectra and X-ray photoelectron spectroscopy (XPS) were conducted. As exhibited in Fig. 1a, the FESEM investigation suggests that the obtained CoNi@NC-CoNi@CNTs-1 displays a cubic sea urchin-like morphology with an evident rough surface compared with CoNi PBAs and CoNi@NC, which is composed of 3D nanocubes and 1D CNTs. Specially, the closer FESEM observation (inset in Fig. 1a) reveals that large quantities of CNTs are stationed on the surface of nanocubes, which results in its rough surface and cubic sea urchin-like morphology. The FESEM observation reveal that only the typical cubic sea urchin-like geometry can be observed in the as-prepared CoNi@NC-CoNi@CNTs-1, implies its high selectivity. Similar to CoNi@NC-CoNi@CNTs-1, the FESEM observations indicate that both the obtained CoNi@NC-CoNi@CNTs-2 (Fig. 1b) and CoNi@NC-CoNi@CNTs-3 (Fig. 1c) also present typical cubic sea urchin-like morphologies, which are seen in large scale in the obtained samples. And the insets of Fig. 1b, c further confirm that the nanocubes are well encircled by large numbers of CNTs. And the contrastive investigation reveals that the number of CNTs surrounding nanocube in the obtained CoNi@NC-CoNi@CNTs MCNCs is gradually enhanced with increasing the pyrolysis temperature from 660 to 860 °C. To further study their microstructures, TEM investigation was also conducted. As provided in Fig. 1d, the typical TEM image verifies the mixed-dimensional cubic sea urchin-like morphology of CoNi@NC-CoNi@CNTs-1, which is composed of 0D innermost nanoparticles, void layer, 3D NC layer and outermost 0D nanoparticles@1D CNTs. The SEM and TEM results suggest the obtained CoNi@NC-CoNi@CNTs-1 is yolk–shell cubic sea urchin-like mixed-dimensional MCNCs. Equally, the TEM observations (Fig. 1e, f) reveal that the as-prepared CoNi@NC-CoNi@CNTs-2 and CoNi@NC-CoNi@CNTs-3 also contain large quantities of 0D nanoparticles and hollow area, 3D cinereous NC layer and dense 0D nanoparticles@1D CNTs, which simultaneously present the typical yolk–shell structure and mixed-dimensional cubic sea urchin-like morphology. Additionally, the TEM investigation further confirms that an increased number of 1D CNTs are observed on the surface of 3D nanocubes from CoNi@NC-CoNi@CNTs-1 to CoNi@NC-CoNi@CNTs-2 and CoNi@NC-CoNi@CNTs-3. And the sizes of nanocubes and numbers of nanoparticles in nanocubes for CoNi@NC-CoNi@CNTs-1, CoNi@NC-CoNi@CNTs-2, and CoNi@NC-CoNi@CNTs-3 are gradually becoming smaller (as shown in Fig. 1g-h), which also indicates that the number of CNTs is increasing.

**Fig. 2:**
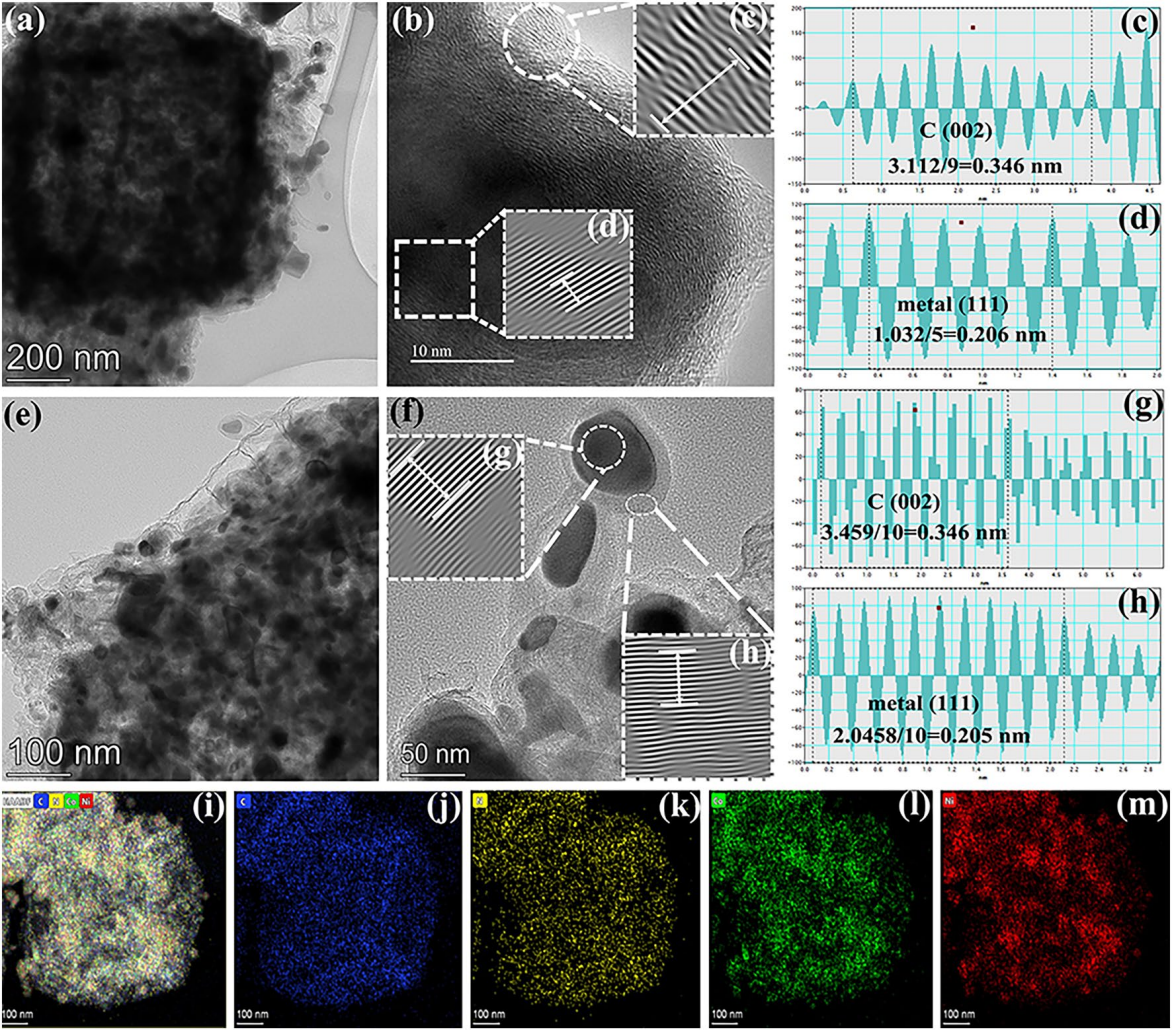
**a-h** TEM, HRTEM, and **i-m** HAADF-STEM and elemental mapping images of CoNi@NC-CoNi@CNTs-1

To further investigate the micromorphology and elemental composition, CoNi@NC-CoNi@CNTs-1 as an example was studied by high-resolution transmission electron microscopy (HRTEM), high-angle annular dark-field scanning transmission electron microscopy (HAADF-STEM) and TEM elemental mapping. As shown in Fig. 2a-f, the enlarged TEM images demonstrate the mixed-dimensional cubic sea urchin-like morphology and yolk–shell structure of CoNi@NC-CoNi@CNT-1. And HRTEM observation (Fig. 2c, d) shows that the cube consists of gray shell and internal nanoparticles, which display the lattice stripes of 0.342 and 0.204 nm spacing in the corresponding HRTEM images (Fig. 2e). Based on the designed experiment and previous result [17], the interplanar spacing of 0.346 and 0.206 nm are well matched with (002) plane of C and (111) plane of CoNi alloy, respectively. Meanwhile, as marked in Fig. 2g, h, HRTEM investigation for the top area of CNTs indicate that the obtained nanoparticle located into CNTs also present an interlayer distance of 0.205 nm, which also corresponds to the (111) crystal plane of CoNi alloy. And multilayer graphitized carbon shell can also be observed around the CoNi nanoparticles, with a spacing of 0.346 nm. According to the HAADF-STEM and elemental mapping images of CoNi@NC-CoNi@CNT-1 (Fig. 2i-m), C, N, Co, Ni, and elements coexist are uniformly dispersed on the cube. Co and Ni elements can be clearly observed at the top of CNTs and interior of cube. According to the obtained characterization results and previous model [27–29], the formation of mixed-dimensional cubic sea urchin-like CoNi@NC-CoNi@CNTs MCNCs can be attributed to the following aspects (Fig. S2): (i) the decomposition and reduction of CoNi PBAs induces the generation of CoNi@NC, and (ii) generated CoNi@NC can be acted as a good catalyst for the growth of CNTs. In general, the obtained FESEM, TEM, FESEM and elemental mapping results confirm that cubic sea urchin-like CoNi@NC-CoNi@CNTs with yolk–shell structure mixed-dimensional MCNCs can be produced in high efficiency through our proposed simple route.

**Fig. 3:**
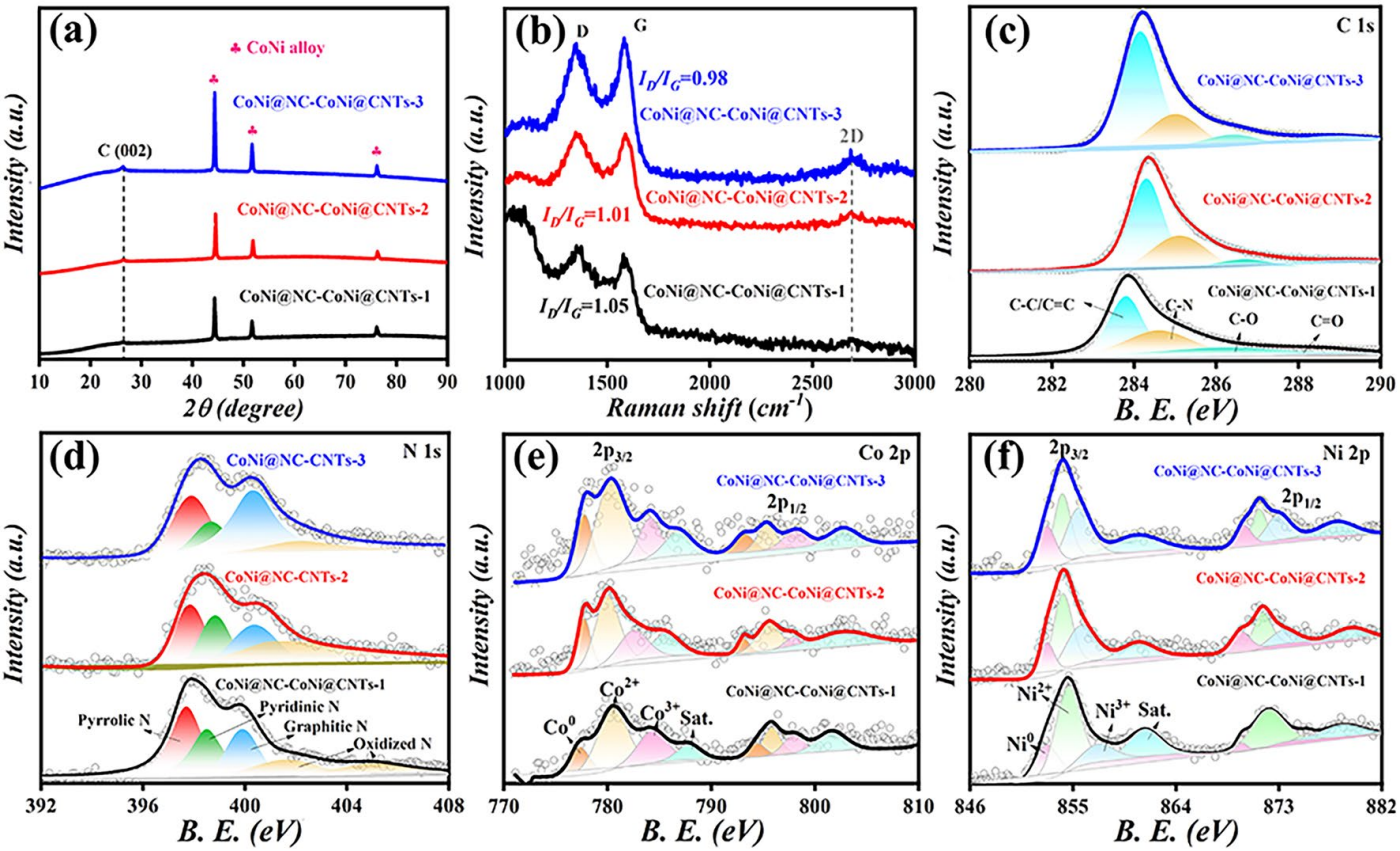
**a** XRD patterns, **b** Raman spectra, **c-f** high-resolution XPS spectra for CoNi@NC-CoNi@CNTs MCNCs

Figure 3 presents the XRD, Raman, and XPS results of CoNi@NC-CoNi@CNTs MCNCs. As presented in Fig. 3a, it can be observed that all the as-prepared CoNi@NC-CoNi@CNTs MCNCs present three strong diffraction peaks at ca. 44.34°, 51.68°, and 76.12°, which can be assigned to the (111), (200), and (220) crystal planes of CoNi alloy [30, 31]. In addition, a diffraction peak appearing at about 26° corresponds to the (002) crystal plane of graphitic carbon. And the obtained CoNi@NC-CoNi@CNTs MCNCs display the gradually obvious carbon diffraction peaks when the pyrolysis temperature increases from 660 to 860 °C, which implies the increased degree of graphitization and/or enhanced content of carbon [3, 11]. As shown in Fig. 3b, two Raman peaks located at ca. 1350 and 1580 cm^−1^ corresponding to D-band (disorder carbon) and G-band (graphitic carbon) can be evidently observed [24]. The *I*_D_/*I*_G_ values for CoNi@NC-CoNi@CNTs-1, CoNi@NC-CoNi@CNTs-2, and CoNi@NC-CoNi@CNTs-3 are 1.05, 1.01, and 0.98, which further confirms the evidently improved graphitization degree of carbon. Meanwhile, with the pyrolysis temperature enhancing from 660 to 860 °C, the obtained CoNi@NC-CoNi@CNTs MCNCs present the increasingly sharp 2D peak (located at 2700 cm^−1^) associated with the graphite layer, which also indicates that CoNi@NC-CoNi@CNTs-3 is highly graphitized, which helps to enhance the dielectric loss. The XPS survey spectra (Fig. S3a) of CoNi@NC-CoNi@CNTs illustrate the existing elements of C 1*s*, N 1*s*, Co 2*p*, and Ni 2*p*, which further confirms the successful production of CoNi@NC-CoNi@CNTs MCNCs. As labeled in Fig. 3c, four split peaks in the C 1 s high-resolution spectra are attributed to the C–C/C = C, C = N, C-O, and C = O, respectively [11, 32]. As provided in Fig. 3d, the peaks of N 1*s* are decomposed into several Gaussian peaks containing pyridinic N (397.9 eV), pyrrolic N (398.9 eV), graphitic N (400.4 eV) and oxidized N (401.8 eV) [27]. As summarized in Fig. S3b, the as-prepared CoNi@NC-CoNi@CNTs present the evidently enhanced content of graphitic N, which is beneficial to boost the conductivity and thereby increasing conduction loss ability [33]. Furthermore, the Co 2*p* and Ni 2*p* high-resolution XPS spectrum (Fig. 3e, f) are deconvoluted into three spin–orbit doublets characteristics of Co^0^, Co^2+^ and Co^3+^, Ni^0^, Ni^2+^ and Ni^3+^ [34, 35]. The presence of Co^0^ and Ni^0^ in the XPS results are consistent with the XRD and TEM outcomes, indicating the successful preparation of CoNi@NC-CoNi@CNTs. Generally, the obtained results demonstrate that the content of CNTs and graphitization degree of carbon for designed CoNi@NC-CoNi@CNTs can be effectively modulated by regulating the pyrolysis temperature, which favors to improve EM parameters and MA performances.

### Tunable MA Performance and Loss Mechanisms of CoNi@NC-CoNi@CNTs MCNCs

**Fig. 4:**
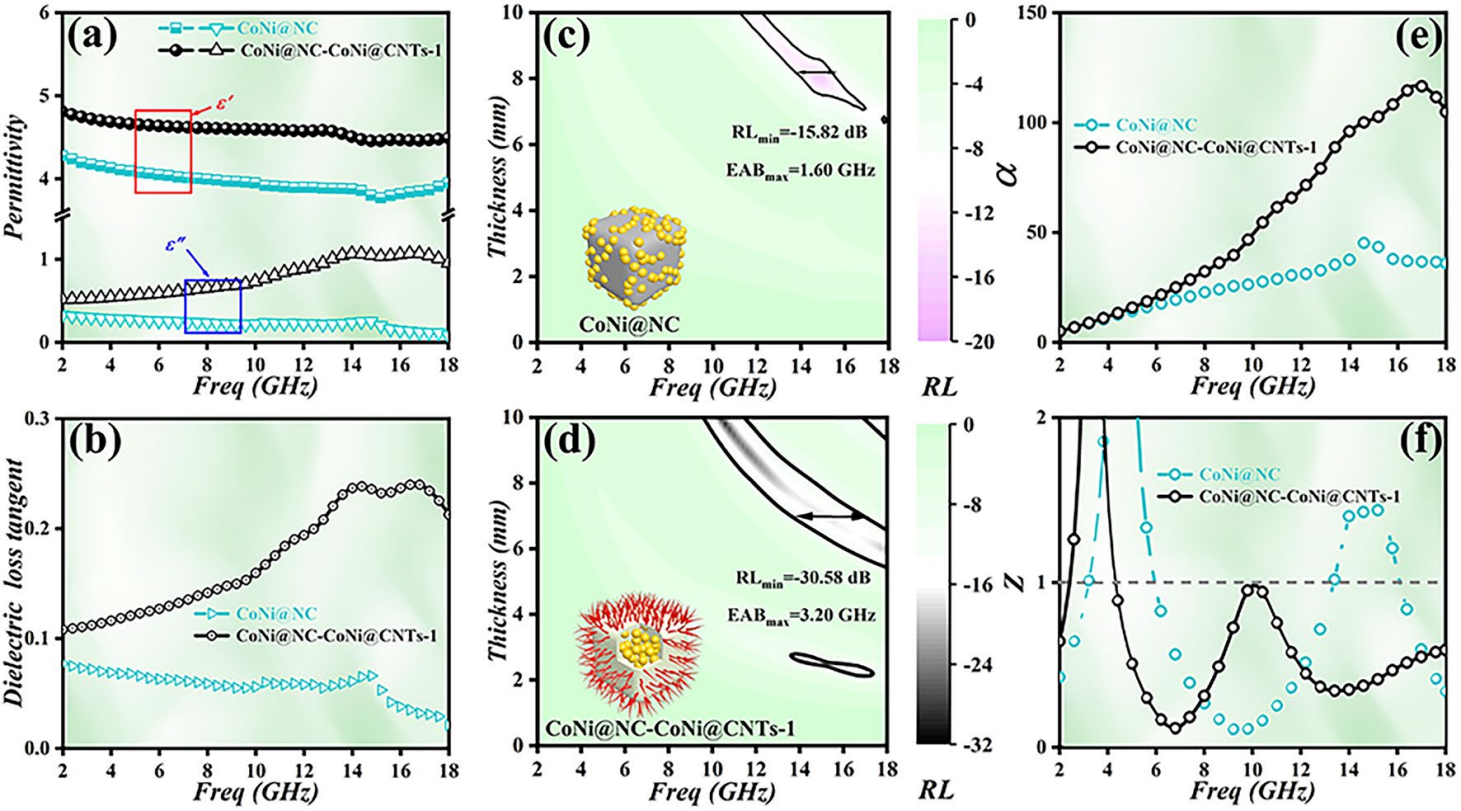
**a** Complex permittivity, **b** dielectric loss tangent values, **c**, **d** RL color maps, **e** attenuation constant and **f** impedance matching characteristics for CoNi@NC and CoNi@NC-CoNi@CNTs-1 PS

For comparison, Fig. 4 provides the complex permittivity, dielectric loss tangent ($\tan \delta_{\varepsilon } = {{\varepsilon^{\prime\prime}} \mathord{\left/ {\vphantom {{\varepsilon^{\prime\prime}} {\varepsilon^{\prime}}}} \right. \kern-0pt} {\varepsilon^{\prime}}}$), RL, attenuation constant ($\alpha$) and $Z$ values for CoNi@NC and CoNi@NC-CoNi@CNTs-1. As presented in Fig. 4a, the $\varepsilon^{\prime}$ and $\varepsilon^{\prime\prime}$ values of CoNi@NC vary in the range of 4.28–3.95 and 0.33–0.08, respectively. Compared to CoNi@NC, the introduction of CNTs results in the enhanced $\varepsilon^{\prime}$ and $\varepsilon^{\prime\prime}$ values of CoNi@NC-CoNi@CNTs-1, which locate in the range of 4.81–4.49 and 0.52–0.95 [36, 37]. As presented in Fig. 4b, the obtained CoNi@NC-CoNi@CNTs-1 exhibits much larger $\tan \delta_{\varepsilon }$ values than CoNi@NC, implying the boosted dielectric loss ability [34, 38]. In light of Eqs. (S1) and (S2), complex permittivity and complex permeability (Fig. S4), their RL values can be acquired. The 2D RL plots (Fig. 4c, d) indicate that CoNi@NC-CoNi@CNTs-1 shows excellent MA performance including *RL*_min_ of -30.58 dB and EAB of 3.20 GHz, which are twice as much as those of CoNi@NC. The comparison results demonstrate that the growth of CNTs and the formation of the hierarchical cubic sea urchin-like yolk–shell structure are beneficial to improve EM and MA performance. As displayed in Fig. 4e, f, the acquired $\alpha$ and $Z$ values reveal that the designed CoNi@NC-CoNi@CNTs-1 display the evidently superior EM wave attenuation and impedance matching properties, which contribute to the boosted MA performance [8, 9, 39].

**Fig. 5:**
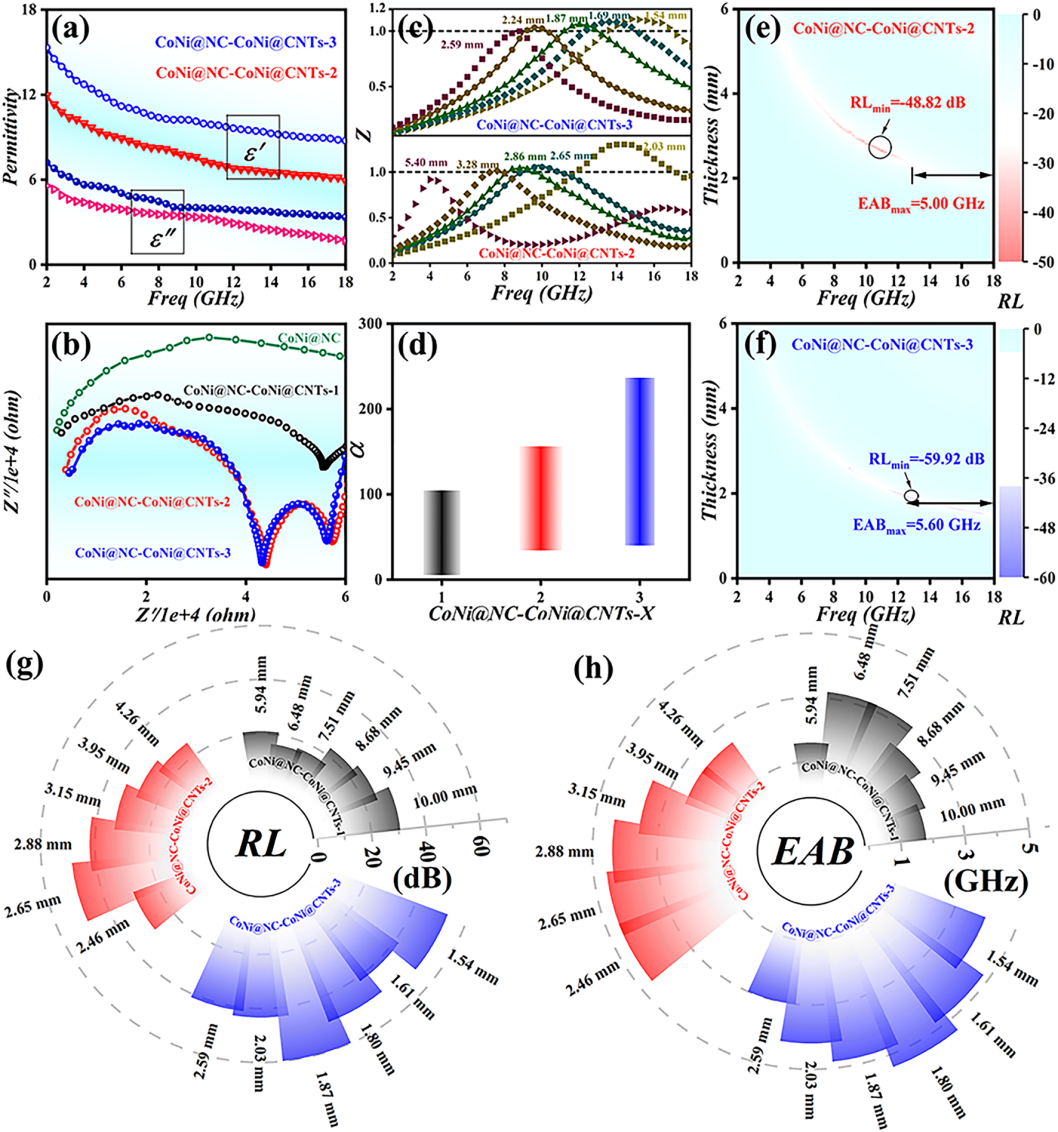
**a** Complex permittivity, **b** Nyquist plots, **c** $Z$ curves, **d** $\alpha$ values, **e**, **f** RL color maps, **g** RL values and **h** EAB values of CoNi@NC-CoNi@CNTs MCNCs

In light of acquired results, the catalytic decomposition of melamine over CoNi@NC results in the formation of yolk–shell CoNi@NC-CoNi@CNTs mixed-dimensional MCNCs, which greatly improve the MA performances compared with CoNi@NC. To further optimize EM and MA performances, CoNi@NC-CoNi@CNTs-2 and CoNi@NC-CoNi@CNTs-3 are also produced. As presented in Fig. 5a, CoNi@NC-CoNi@CNTs-3 displays the higher $\varepsilon^{\prime}$ and $\varepsilon^{\prime\prime}$ values than those of CoNi@NC-CoNi@CNTs-2, which is ascribed to the enhanced CNTs content. And Nyquist plots (Fig. 5b) show their following relationship of conductivity: CoNi@NC < CoNi@NC-CoNi@CNTs-1 < CoNi@NC-CoNi@CNTs-2 < CoNi@NC-CoNi@CNTs-3, which implies their boosted electrical conductivity. The obtained results suggest that the growth of CNTs and enhanced content of CNTs helps to enhance the electrical conductivity, which are consistent with the acquired $\varepsilon^{\prime\prime}$ values. As given in Fig. 5c, the contrastive outcomes reveal that CoNi@NC-CoNi@CNTs-3 displays the best impedance matching characteristic and CoNi@NC-CoNi@CNTs-1 presents the worst one. And their $\alpha$ values (Fig. 5d) are as follows: CoNi@NC-CoNi@CNTs-3 > CoNi@NC-CoNi@CNTs-2 > CoNi@NC-CoNi@CNTs-1 > CoNi@NC. The $\alpha$ and $Z$ values reveal that the CNTs introduction and enhanced CNTs content greatly improve the EM wave attenuation and impedance matching performances. As displayed in Fig. 5e, f, CoNi@NC-CoNi@CNTs-2 exhibits good MA performances with the *RL*_min_ value of -48.82 dB at the matching thickness (*d*_m_) of 2.65 mm and frequency of 11 GHz, and the EAB value can reach 5 GHz (from 12 to 17 GHz). Apparently, CoNi@NC-CoNi@CNTs-3 displays satisfactory MA performances with a *RL*_min_ of -59.92 dB (*d*_m_ = 1.87 mm), and EAB of 5.6 GHz at 1.71 mm. As summarized in Fig. 5g, h, the contrastive results reveal that the designed CoNi@NC-CoNi@CNTs MCNCs present the evidently boosted MA performance with increasing the pyrolysis temperature. This indicates that the content of CNTs and carbon graphitization degree can effectively adjust the *RL*, EAB and *d*_m_ of CoNi@NC-CoNi@CNTs MCNCs.

**Fig. 6:**
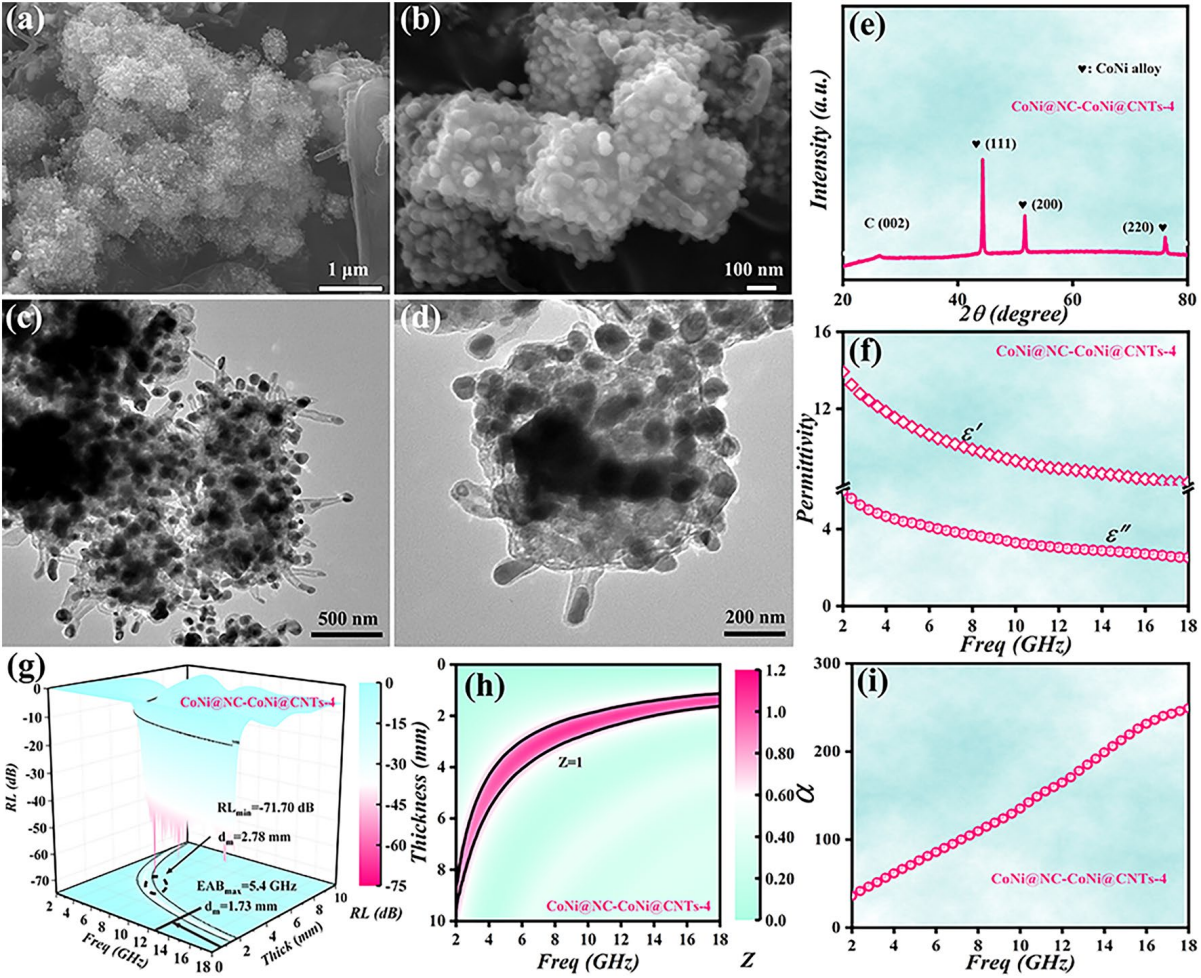
**a-d** SEM and TEM images, **e** XRD pattern, **f** complex permittivity, **g** 3D RL color maps, **h** $Z$ color maps and **i** $\alpha$ curves of CoNi@NC-CoNi@CNTs-4

As mentioned in experimental section, different pyrolysis time (4 h) at 660 °C was also conducted to produce CoNi@NC-CoNi@CNTs MCNCs labeled as CoNi@NC-CoNi@CNTs-4. Similar to CoNi@NC-CoNi@CNTs-1, SEM and TEM investigations (Fig. 6a-d) reveal that the obtained CoNi@NC-CoNi@CNTs-4 also shows a mixed-dimensional cubic sea urchin-like morphology, which consists of CoNi@NC nanocubes and large number of CoNi@CNTs growing around the nanocubes. Compared with CoNi@NC-CoNi@CNTs-1, these investigations reveal that much more 1D CNTs are wrapped around 3D nanocubes in CoNi@NC-CoNi@CNTs-4, which suggests that the prolonged pyrolysis time helps the growth of CNTs [8, 10]. To understand its chemical composition and bonding state, it was similarly characterized by XPS. As shown in Fig. S5, CoNi@NC-CoNi@CNTs-4 also exhibits the similar valence state and elemental composition as CoNi@NC-CoNi@CNTs-1, CoNi@NC-CoNi@CNTs-2 and CoNi@NC-CoNi@CNTs-3. Similarly, three diffraction peaks (Fig. 6e) assignable to the (111), (200), and (220) crystal planes of CoNi and broad peak corresponding to the (002) plane of graphitic carbon can also be clearly detected over the CoNi@NC-CoNi@CNTs-4, further confirm the obtained sample is cubic sea urchin-like yolk–shell CoNi@NC-CoNi@CNTs MCNCs. As shown in Fig. 6f, the $\varepsilon^{\prime}$ and $\varepsilon^{\prime\prime}$ of CoNi@NC-CoNi@CNTs-4 locate in the range of 13.92–8.18, and 5.81–2.51, which are much higher than those of CoNi@NC-CoNi@CNTs-1. In light of acquired results, the evidently enhanced $\varepsilon^{\prime}$ and $\varepsilon^{\prime\prime}$ values of CoNi@NC-CoNi@CNTs-4 mainly originate from the increased contents of CNTs. And the acquired 3D RL plot (Fig. 6g) suggests that the CoNi@NC-CoNi@CNTs-4 exhibits a *RL*_min_ value of -71.70 dB at the thickness of 2.78 mm, and EAB of 5.4 GHz (from 12.6 to 18 GHz). And the obtained CoNi@NC-CoNi@CNTs-4 also displays very excellent MA performance in the whole tested frequency region (Fig. S6). Additionally, as presented in Fig. 6h, i, the CoNi@NC-CoNi@CNTs-4 presents very outstanding EM wave attenuation and impedance matching performances, which contribute to its outstanding MA properties. Based on the reported models and our obtained results [40, 41], the main EM wave loss pathways for CoNi@NC-CoNi@CNTs MCNCs mainly originate from the following aspects (Fig. S7). (i) Polar groups, N doping and other defects acting as polarization centers generate dipole polarization [42]. (ii) CNTs surrounding nanocubes provides abundant channels for electron transmission and enhances conduction loss (Fig. S8a) [40]. (iii) Multiple heterogeneous interfaces induce rich interface polarization effect (Fig. S8b) [43]. (iv) Excellent synergistic effect makes the CoNi@NC-CoNi@CNTs display the outstanding attenuation ability of EM wave and impedance matching characteristic.

### Multifunctionality of CoNi@NC-CoNi@CNTs Mixed-Dimensional MCNCs Suitable for Special Environment

#### Light–Thermal–Electric Energy Cycle

**Fig. 7:**
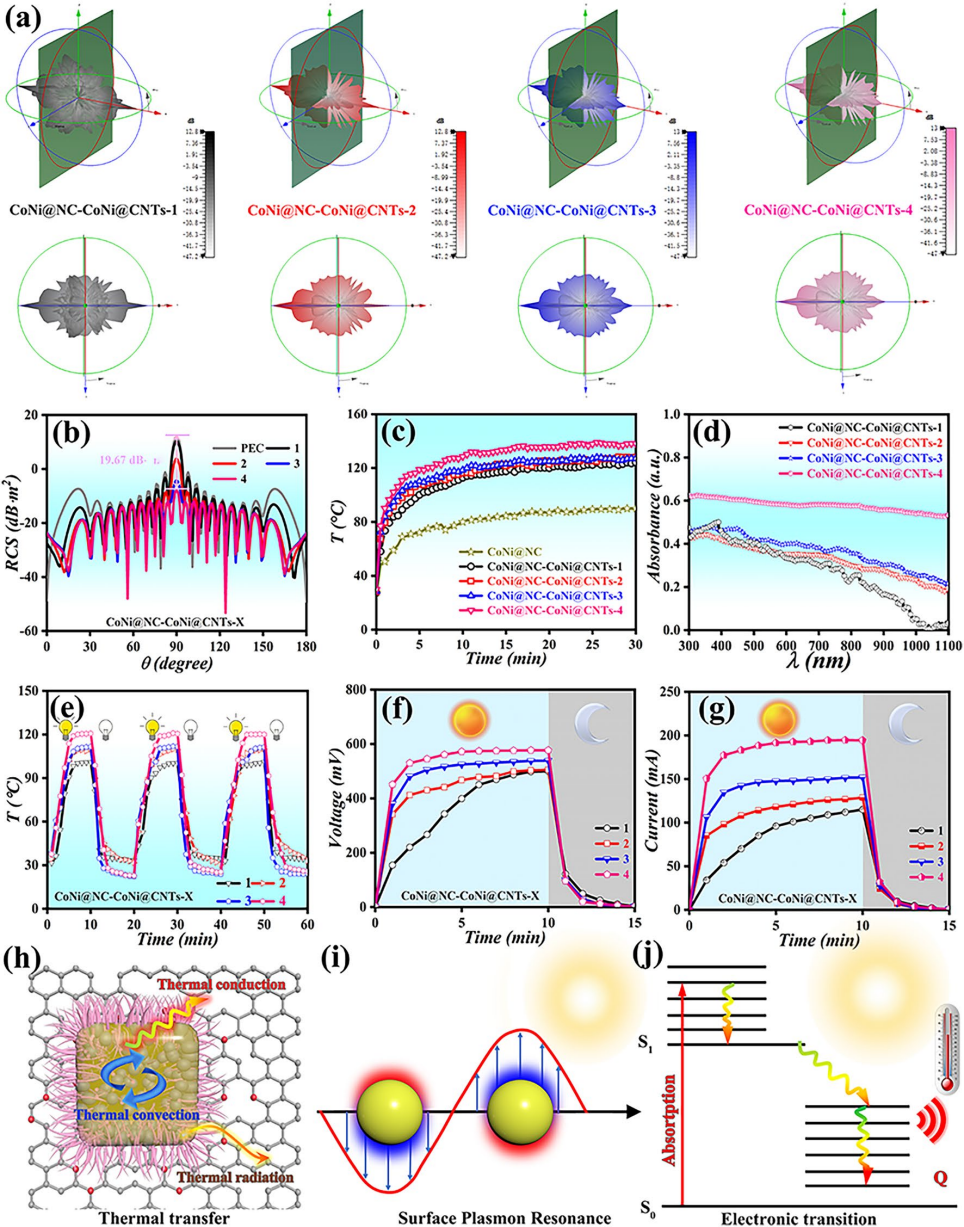
**a**, **b** 3D RCS simulation and simulated RCS values at 0–180° incident angle, **c** T-t curves, **d **UV–vis-NIR absorption spectra, **e** temperature change curves of heating and cooling processes, **f** open-circuit voltage, **g** short-circuit currents, **h** thermal transfer mechanism, **i,** **j** photothermal conversion mechanism for CoNi@NC-CoNi@CNTs MCNCs

In order to evaluate the actual microwave dissipation capacity in the far-field conditions, the Radar cross section (RCS) values for CoNi@NC-CoNi@CNTs covered with PEC model were acquired by CST Studio Suite simulation. Figures 7a and S9 depict the 3D radar wave scattering signals of CoNi@NC-CoNi@CNTs MCNCs and pure PEC. It is distinct that the designed CoNi@NC-CoNi@CNTs MCNCs present obviously weak scattering intensity compared with pure PEC model. Besides, CoNi@NC-CoNi@CNTs-4 displays the weakest scattering intensity than other CoNi@NC-CoNi@CNTs MCNCs, indicating that the CoNi@NC-CoNi@CNTs-4 possesses the lowest RCS value. The detailed RCS values in the 0–180° angle range are presented in Fig. 7b. The relationship of RCS value is as bellow: pure PEC > CoNi@NC-CoNi@CNTs-1 > CoNi@NC-CoNi@CNTs-2 > CoNi@NC-CoNi@CNTs-3 > CoNi@NC-CoNi@CNTs-4, suggesting that CoNi@NC-CoNi@CNTs can effectively reduce surface reflection. Notably, the RCS value of CoNi@NC-CoNi@CNTs-4 is -53.23 dB m^2^ at 124° pulse width angle, and the RCS reduction covers almost all pulse width angles. Compared to reported composites [38, 44, 45], CoNi@NC-CoNi@CNTs-4 show lower RCS value, which not only effectively dissipates the EM wave, but also reduces the radar scattering intensity [46, 47]. To investigate their thermal conductivity performances, the surface temperatures of sample (1.68 mm thickness) were measured after exposure to a hotplate for 30 min. As shown in Figs. 7c and S10a, except for CoNi@NC, the surface temperatures of CoNi@NC-CoNi@CNTs are above 100°C, indicating their efficient heat transfer performances and temperature stability. When the coating absorbing material is also a photothermal material, it can provide energy when military aircraft operate or out of service, thus maximizing the energy benefits. The light absorption curves (Fig. 7d) show that the CoNi@NC-CoNi@CNTs MCNCs display greatly enhanced light absorption capabilities in the whole wavelength range (300–1100 nm) with modulating the pyrolysis temperature and time, which may be due to their enhanced graphitization degree and color (black) [18]. And the sea urchin-like yolk–shell structure CoNi@NC-CoNi@CNTs further allows the incident electric field to strongly penetrate the contraction geometry effect and contributes to the broadband absorption [48, 49].

To explore the photothermal conversion properties, the surface temperatures of CoNi@NC-CoNi@CNTs was tested under simulated sunlight irradiation. As illustrated in Figs. 7e and S10b, all the MCNCs display the stable T-t change curves after three repetitions, implies their excellent and stable photothermal conversion properties. And their excellent properties are mainly due to the interaction of CoNi nanoparticles and carbon, and the existence of 3D sea urchin-like yolk–shell structure promoting the non-radiative compounding, which effectively releases thermal energy and promotes the photothermal effect [50, 51]. In general, the as-prepared CoNi@NC-CoNi@CNTs MCNCs have good photothermal characteristics and thermal transfer efficiency, which can be used as an excellent candidate photothermal material. To achieve energy recycling, a solar thermoelectric generator (STEG) was constructed by placing a thermoelectric module (SP1848-27,145 SA) between the cooling plate and CoNi@NC-CoNi@CNTs (Fig. S10c). From the sample to thermoelectric module, the thermal side temperature of thermoelectric module gradually increases as the light time increases. Correspondingly, after 10 min of illumination, the V_OC_ and I_SC_ of STEG are 500 mV and 115 mA, 505 mV and 128.6 mA, 539 mV, and 151.9 mA, 577 mV and 194.5 mA, respectively (Fig. 7f, g). Therefore, the integrated thermal power module of MCNCs in this study provides potential ideas for thermal energy collection and conversion. According to the obtained results, the yolk–shell and 3D cubic structure connected by CNTs enhance the thermal radiation, thermal conduction and thermal convection (Fig. 7h) [2, 10, 52]. As presented in Fig. 7i, electrons on the surface of CoNi nanoparticles absorb photons and generate thermal electrons, resulting light-to-heat conversion (surface plasmon resonance effects) [53]. Furthermore, the electrons of carbon frames and CNTs can absorb the energy of photons, putting the electrons in an excited state. The electrons in the excited state will decay back to the ground state at any time due to their instability, which process photothermal effect (Fig. 7j) [54, 55]. In general, the designed CoNi@NC-CoNi@CNTs MCNCs can absorb EM waves and solar energy, and convert them into heat energy to be used, achieving real-time and efficient heat exchange.

#### Hydrophobicity and Anti-Corrosion Performance

**Fig. 8:**
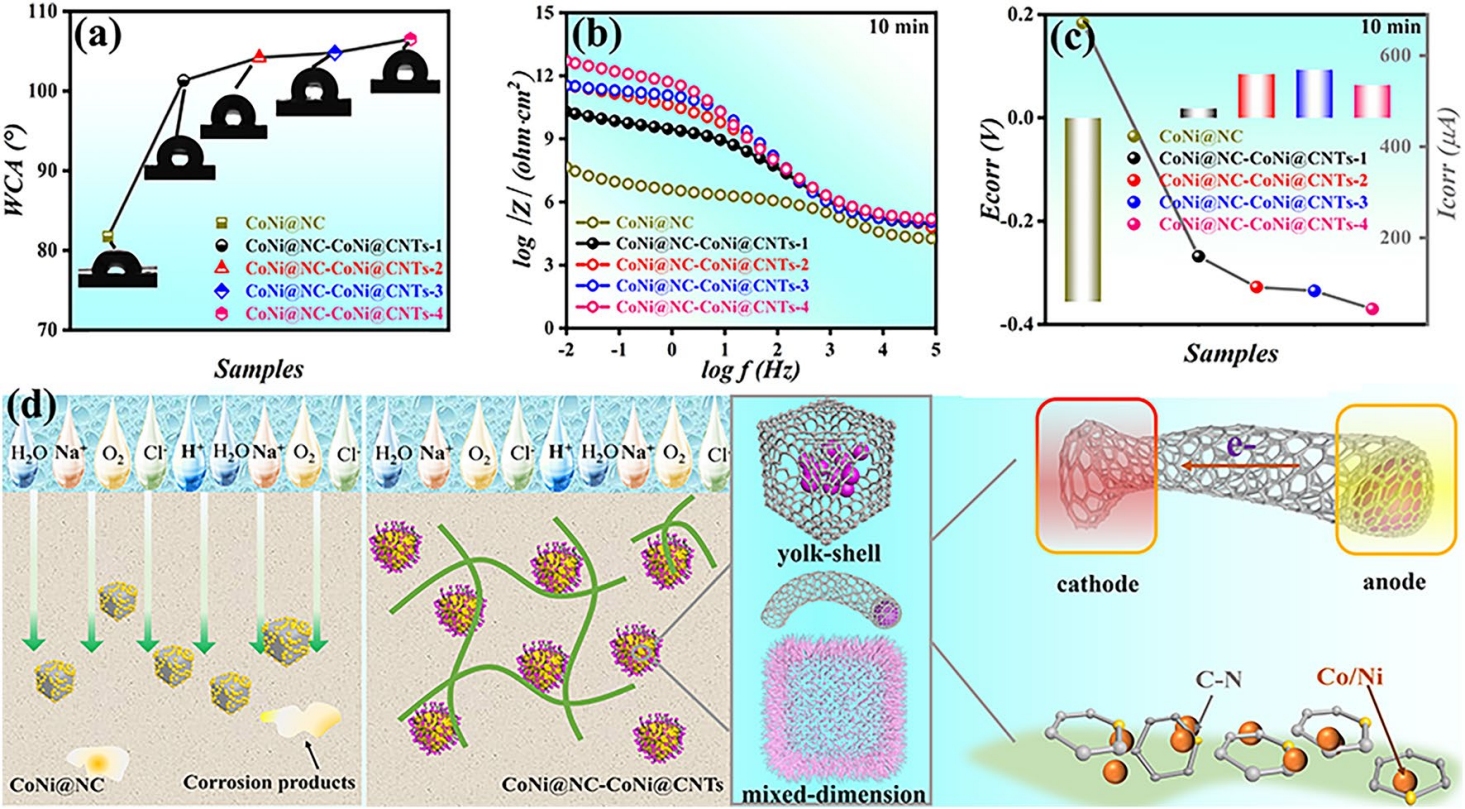
**a** WCAs, **b** impedance modulus curves, **c** *E*_corr_ and *I*_corr_, and **d** schematic diagrams of anti-corrosion mechanisms for CoNi@NC-CoNi@CNTs MCNCs

For submarines working in a deep ocean, the shell protection layer needs to have a certain corrosion prevention and bacteriostatic effect to ensure their normal operation. Figure 8a illustrates the water contact angle (WCA) of CoNi@NC (81.7°) is much smaller than ones of CoNi@NC-CoNi@CNTs (WCA > 100°), indicating that the growth of CNTs helps to enhance the hydrophobicity of mixed-dimensional MCNCs [56]. According to the previous results [54, 57], the enhanced hydrophobicity can be attributed to the formation of CNTs and increased surface roughness of CoNi@NC-CoNi@CNTs. The anti-corrosion ability of CoNi@NC-CoNi@CNTs were immersed in 3.50 wt% NaCl solution (10 min) for simulating the ocean environment. The open-circuit potential (OCP) test results (Fig. S11a) indicate that CoNi@NC-CoNi@CNTs MCNCs are greater than CoNi@NC. As illustrated in Fig. 8b, the impedance modulus |Z|_0.01 Hz_ values are 7.66 (CoNi@NC), 10.29 (CoNi@NC-CoNi@CNTs-1), 11.54 (CoNi@NC-CoNi@CNTs-2), 11.54 (CoNi@NC-CoNi@CNTs-3), and 12.71 (CoNi@NC-CoNi@CNTs-4) Ω cm^2^, respectively. Meanwhile, the corrosion potential (E_corr_) and corrosion current density (I_corr_) can be obtained according to the Tafel curve (Fig. S11b). As summarized in Fig. 8c, compared with CoNi@NC (-0.36 V), the E_corr_ values of all CoNi@NC-CoNi@CNTs are positive, indicating that CoNi@NC-CoNi@CNTs have certain barrier performance. And the statistical I_corr_ values suggest the worst corrosion resistance of CoNi@NC and best corrosion resistance of CoNi@NC-CoNi@CNTs-4. To assess the stability, CoNi@NC-CoNi@CNTs-3 and CoNi@NC-CoNi@CNTs-4 were immersed in the test solution for 7 days. Their OCP and |Z|_0.01 Hz_ results (Fig. S12) reveal the long-time corrosion resistance stabilities of CoNi@NC-CoNi@CNTs MCNCs, which further confirm that CNTs and hierarchical cubic sea urchin-like yolk–shell structure strengthen the corrosion resistance. Additionally, Fig. S13 provides the complex permittivity, RL and EAB values of CoNi@NC-CoNi@CNTs-3 and CoNi@NC-CoNi@CNTs-4 after immersed in the test solution for 7 days. The comparison results show that the RL and EAB values of CoNi@NC-CoNi@CNTs-3 and CoNi@NC-CoNi@CNTs-4 are only slightly changed before and after soaking, again demonstrating their excellent MA performances and stabilities due to unique microstructure and rationally constructed chemical components. Besides, compared with other MCNCs, the customized CoNi@NC-CoNi@CNTs MCNCs show impressive comprehensive MA performances (Fig. S14). According to the results and the analysis of previous literature [13, 32, 35], the corrosion resistance of CoNi@NC-CoNi@CNTs MCNCs mainly benefits from the following aspects (Fig. 8d): Firstly, hydrophobic property reduces the direct contact between corrosive medium and the surface of CoNi@NC-CoNi@CNTs [13]. Secondly, the special structure provides effective encapsulation and protection for CoNi nanoparticles, which effectively prevent CoNi nanoparticles from direct contact with corrosion medium, thus great reducing the possibility of corrosion reaction [58]. Moreover, mixed-dimensional cubic yolk–shell structure fully plays the role of “physical barrier” and the “maze effect” to delay the penetration rate of corrosion [59]. Lastly, the primary cell effect may be formed between CoNi nanoparticles and CNTs in CoNi@NC-CoNi@CNTs, with CoNi nanoparticles serving as the anode and CNTs serving as the cathode. Due to the good conductivity and stability of CNTs, it can serve as an electron transport channel to quickly transfer the electrons generated by the anode to the cathode, thus slowing down the corrosion rate of the anode [57, 60]. In light of acquired results, the fantastic long-time corrosion performances of CoNi@NC-CoNi@CNTs mixed-dimensional MCNCs can be attributed to the growth of CNTs and formed 3D cubic sea urchin-like yolk–shell structure, which effectively suppresses the corrosion of CoNi nanoparticles.

#### Antibacterial Performance

**Fig. 9:**
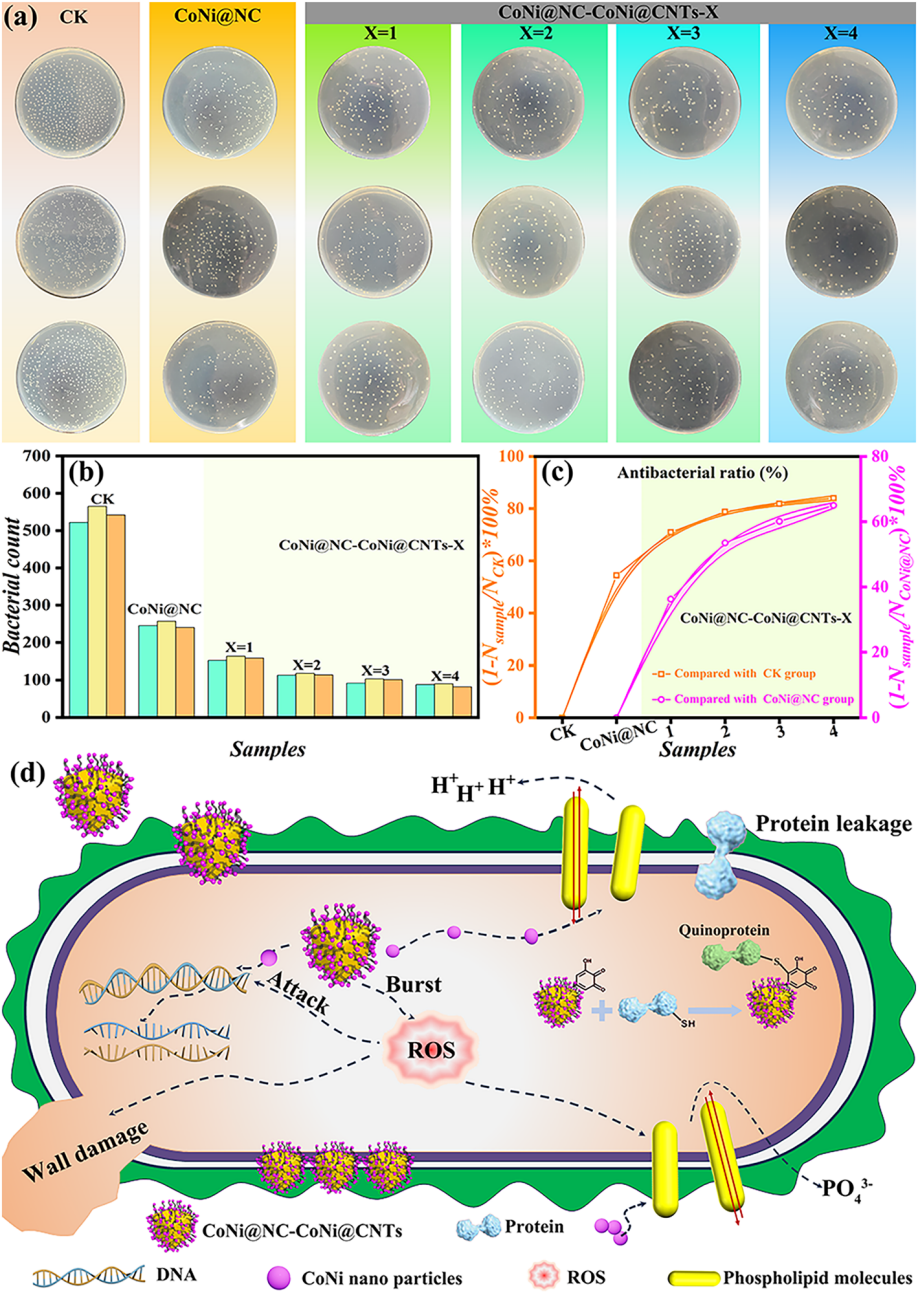
**a** E. coli colony distribution on agar plate of different materials, **b** bacterial count, **c** antibacterial ratio and **d** antibacterial schematic illustration for CoNi@NC-CoNi@CNTs MCNCs

Considering the complexity of real environment, the microorganisms in the environment will have some harm to the material/human body, so it is necessary to investigate the antibacterial property of materials. As shown in Fig. 9a, Escherichia coli (E. coli) multiplies rapidly in sterile solution (CK, blank control group). And the colonies are evenly dispersed in the petri dishes after culture at 37 °C for 18 h. After statistical and calculation comparison (Fig. 9b, c), the number of bacteria is CK > CoNi@NC > CoNi@NC-CoNi@CNTs-1 > CoNi@NC-CoNi@CNTs-2 > CoNi@NC-CoNi@CNTs-3 > CoNi@NC-CoNi@CNTs-4, which indicates that increasing the annealing temperature and extending the annealing time both help to enhance their antibacterial performances. Compared with CK group, the values of average antibacterial rate $\left( \left( 1 - N_{\text{sample}} / N_{CK}  \right) \times 100 \% \right)$ for CoNi@NC, CoNi@NC-CoNi@CNTs-1, CoNi@NC-CoNi@CNTs-2, CoNi@NC-CoNi@CNTs-3, and CoNi@NC-CoNi@CNTs-4 are 54.43%, 70.96%, 78.81%, 81.84%, and 84.03%. Equally, as summarized in Fig. 9c, CoNi@NC-CoNi@CNTs MCNCs display the much higher average antibacterial rates than CoNi@NC. The comparison results prove that the growth of CNTs and the formation of hierarchical cubic sea urchin-like yolk–shell structure are beneficial for enhanced antibacterial properties [61, 62]. In addition, compared with recently representative works [63–67], the CoNi@NC-CoNi@CNTs MCNCs also show good antibacterial performance (Table S2).

Combining the obtained results with previously reported models [15], the main antibacterial mechanisms of hierarchical cubic sea urchin-like yolk–shell CoNi@NC-CoNi@CNTs MCNCs are depicted in Fig. 9d. It can be summarized into the following aspects: (I) CoNi@NC-CoNi@CNTs with hierarchical cubic sea urchin-like structure greatly increases the chance of contact bacteria and boosts bacterial capture efficiency [62, 68]. (II) Due to electrostatic adsorption, CoNi nanoparticles attack the phospholipid bilayer, thereby destroying bacterial membrane permeability [69]. (III) CNTs can penetrate the cell membrane due to high surface activity, resulting in cytoplasmic efflux and cell death [70]. (IV) CoNi and CNTs generate reactive oxygen species (ROS), which bind to DNA/RNA, disrupting the structure of bacterial nucleic acid molecules and inactivating the bacteria [70, 71]. (V) CoNi@NC-CoNi@CNTs readily oxidize to form electron-deficient semiquinones that bind to sulfhydryl residues on proteins, forming quinoproteins (quinone–protein conjugates), thereby killing bacteria [69]. In conclusion, the prepared CoNi@NC-CoNi@CNTs MCNCs is a multifunctional material integrating hydrophobic, corrosion resistant, bacteriostatic and other properties, suitable for the terrestrial and ocean environments.

## Conclusions

In summary, a series of novel cubic sea urchin-like yolk–shell CoNi@NC-CoNi@CNTs mixed-dimensional MCNCs were prepared through a PBAs derived and catalytical chemical vapor deposition strategy. The obtained results demonstrated that the PBAs derived CoNi nanoparticles effectively catalyzed the growth of CNTs, which could be modulated by controlling the pyrolysis temperature and/or time. Moreover, the generated CNTs not only boosted the polarization loss, but also established an interconnected conductive network to efficiently dissipate microwaves. Therefore, the designed CoNi@NC-CoNi@CNTs mixed-dimensional MCNCs presented excellent attenuation capability and good impedance matching characteristics, resulting in extraordinary comprehensive MA performances. Benefiting from the biotoxicity of magnetic metal nanoparticles, high dispersibility of CNTs and hierarchical yolk–shell mixed-dimensional structure, the acquired CoNi@NC-CoNi@CNTs mixed-dimensional MCNCs possessed excellent antibacterial properties, corrosion resistance, photothermal effect and further realized the recycling of three kinds of energy: light, heat and electricity. Therefore, this finding significantly enhanced the adaptability of CoNi@NC-CoNi@CNTs MCNCs in complex and changing environments, and further broadening their potential applications in various fields.

## Supplementary Information

Below is the link to the electronic supplementary material.

Supplementary file1 (DOCX 19649 KB)

## References

[1] J. Cheng, Y. Jin, J. Zhao, Q. Jing, B. Gu et al., From VIB- to VB-group transition metal disulfides: structure engineering modulation for superior electromagnetic wave absorption. Nano-Micro Lett. **16**, 29 (2023). 10.1007/s40820-023-01247-7 10.1007/s40820-023-01247-7 PMC10667208 37994956

[2] M. He, X. Zhong, X. Lu, J. Hu, K. Ruan et al., Excellent low-frequency microwave absorption and high thermal conductivity in polydimethylsiloxane composites endowed by *Hydrangea*-like CoNi@BN heterostructure fillers. Adv. Mater. **36**, 2410186 (2024). 10.1002/adma.202410186 10.1002/adma.202410186 39380425

[3] X. Zhong, M. He, C. Zhang, Y. Guo, J. Hu et al., Heterostructured BN@Co-C@C endowing polyester composites excellent thermal conductivity and microwave absorption at C band. Adv. Funct. Mater. **34**, 2313544 (2024). 10.1002/adfm.202313544

[4] L. Zhou, P. F. Hu, M. Bai, N. Leng. B. Cai et al., Harnessing the electronic spin states of single atoms for precise electromagnetic modulation. Adv. Mater. 2418321 (2024). 10.1002/adma.202418321 10.1002/adma.202418321 39726342

[5] B.-X. Wang, C. Xu, G. Duan, W. Xu, F. Pi Review of broadband metamaterial absorbers: from principles, design strategies, and tunable properties to functional applications. Adv. Funct. Mater. **33**, 2213818 (2023). 10.1002/adfm.202213818

[6] P. Huang, W.-Q. Han, Recent advances and perspectives of lewis acidic etching route: an emerging preparation strategy for MXenes. Nano-Micro Letters **15**, 68 (2023).. 10.1007/s40820-023-01039-z 10.1007/s40820-023-01039-z PMC10014646 36918453

[7] M.-X. Sun, W.-Q. Cao, P.-Y. Zhu, Z.-M. Xiong, C.-C. Chen et al., Thermally tailoring magnetic molecular sponges through self-propagating combustion to tune magnetic-dielectric synergy toward high-efficiency microwave absorption and attenuation. Adv. Compos. Hybrid Mater. **6**, 54 (2023). 10.1007/s42114-023-00629-0

[8] S. Wang, X. Zhang, S. Hao, J. Qiao, Z. Wang et al., Nitrogen-doped magnetic-dielectric-carbon aerogel for high-efficiency electromagnetic wave absorption. Nano-Micro Lett. **16**, 16 (2023). 10.1007/s40820-023-01244-w 10.1007/s40820-023-01244-w PMC10656410 37975962

[9] C. Li, D. Li, S. Zhang, L. Ma, L. Zhang et al., Interface engineering of titanium nitride nanotube composites for excellent microwave absorption at elevated temperature. Nano-Micro Lett. **16**, 168 (2024). 10.1007/s40820-024-01381-w 10.1007/s40820-024-01381-w PMC10994892 38573346

[10] J. Wang, Z. Miao, K. Gao, Z. Li, X. Zhang et al., Integration of heterogeneous interfaces and multi-dimensional encapsulation structure in Fe_2_N@CNTs enabling highly efficient thermal management and microwave absorption. Adv. Funct. Mater. **34**, 2408696 (2024). 10.1002/adfm.202408696

[11] B. Li, Z. Ma, J. Xu, X. Zhang, Y. Chen et al., Regulation of impedance matching and dielectric loss properties of N-doped carbon hollow nanospheres modified with atomically dispersed cobalt sites for microwave energy attenuation. Small **19**, e2301226 (2023). 10.1002/smll.202301226 36974608 10.1002/smll.202301226

[12] B. Zhao, Z. Yan, L. Liu, Y. Zhang, L. Guan et al., A liquid-metal-assisted competitive galvanic reaction strategy toward indium/oxide Core-Shell nanoparticles with enhanced microwave absorption. Adv. Funct. Mater. **34**, 2314008 (2024). 10.1002/adfm.202314008

[13] Z. Shi, H. Zeng, Y. Yuan, N. Shi, L. Wen et al., Constructing superhydrophobicity by self-assembly of SiO_2_@Polydopamine core-shell nanospheres with robust oil-water separation efficiency and anti-corrosion performance. Adv. Funct. Mater. **33**, 2213042 (2023). 10.1002/adfm.202213042

[14] S. Hao, H. Han, Z. Yang, M. Chen, Y. Jiang et al., Recent advancements on photothermal conversion and antibacterial applications over MXenes-based materials. Nano-Micro Lett. **14**, 178 (2022). 10.1007/s40820-022-00901-w 10.1007/s40820-022-00901-w PMC9402885 36001173

[15] K. Song, Y.-T. Pan, J. Zhang, P. Song, J. He et al., Metal–organic frameworks–based flame-retardant system for epoxy resin: a review and prospect. Chem. Eng. J. **468**, 143653 (2023). 10.1016/j.cej.2023.143653

[16] X. Zhuang, S. Zhang, Y. Tang, F. Yu, Z. Li et al., Recent progress of MOF/MXene-based composites: synthesis, functionality and application. Coord. Chem. Rev. **490**, 215208 (2023). 10.1016/j.ccr.2023.215208

[17] W. Xiao, M. Cheng, Y. Liu, J. Wang, G. Zhang et al., Functional metal/carbon composites derived from metal–organic frameworks: insight into structures, properties, performances, and mechanisms. ACS Catal. **13**, 1759–1790 (2023). 10.1021/acscatal.2c04807

[18] T. De Villenoisy, X. Zheng, V. Wong, S.S. Mofarah, H. Arandiyan et al., Principles of design and synthesis of metal derivatives from MOFs. Adv. Mater. **35**, 2210166 (2023). 10.1002/adma.202210166 10.1002/adma.202210166 36625270

[19] Q. Li, Q. Li, Z. Wang, X. Zheng, S. Cai et al., Recent advances in hierarchical porous engineering of MOFs and their derived materials for catalytic and battery: methods and application. Small **20**, 2303473 (2024). 10.1002/smll.202303473 10.1002/smll.202303473 37840383

[20] A. Sudharshan Reddy, S.P. Pooja Sharda, A.S. Nehra, Advanced strategies in MOF-based mixed matrix membranes for propylene/propane separation: a critical review. Coord. Chem. Rev. **498**, 215435 (2024). 10.1016/j.ccr.2023.215435

[21] T. Wei, Y. Zhou, C. Sun, X. Guo, S. Xu et al., An intermittent lithium deposition model based on CuMn-bimetallic MOF derivatives for composite lithium anode with ultrahigh areal capacity and current densities. Nano Res. **17**, 2763–2769 (2024). 10.1007/s12274-023-6187-8

[22] L. Wang, T. Chen, Y. Cui, J. Wu, X. Zhou et al., Rational design of environmentally friendly carbon nanotube embedded artificial vesicle-structured photocatalysts for organic pollutants degradation. Adv. Funct. Mater. **34**, 2313653 (2024). 10.1002/adfm.202313653

[23] S. Nam, C. Park, S.-H. Sunwoo, M. Kim, H. Lee, M. Lee, D.-H. Kim, Soft conductive nanocomposites for recording biosignals on skin. Soft Sci. **3**, 28 (2023).. 10.20517/ss.2023.19

[24] J. Xiao, B. Zhan, M. He, X. Qi, X. Gong, Jing-Liang Yang, Yunpeng Qu, Junfei Ding, Wei Zhong, Junwei Gu, Interfacial polarization loss improvement induced by the hollow engineering of necklace‐like PAN/carbon nanofibers for boosted microwave absorption. Adv. Func. Mater. 2316722 (2024). 10.1002/adfm.202316722

[25] J. Yang, H. Wang, Y. Zhang, H. Zhang, J. Gu, Layered structural PBAT composite foams for efficient electromagnetic interference shielding. Nano-Micro Lett. **16**, 31 (2023). 10.1007/s40820-023-01246-8 10.1007/s40820-023-01246-8 PMC10667195 37994969

[26] S. Wang, W. Huo, H. Feng, Z. Xie, J.K. Shang et al., Enhancing oxygen evolution reaction performance in Prussian blue analogues: triple-play of metal exsolution, hollow interiors, and anionic regulation. Adv. Mater. **35**, e2304494 (2023). 10.1002/adma.202304494 37473821 10.1002/adma.202304494

[27] J. Chen, Y. Ha, R. Wang, Y. Liu, H. Xu et al., Inner co synergizing outer Ru supported on carbon nanotubes for efficient pH-universal hydrogen evolution catalysis. Nano-Micro Lett. **14**, 186 (2022). 10.1007/s40820-022-00933-2 10.1007/s40820-022-00933-2 PMC9475008 36104459

[28] X. Shi, Z. Xu, C. Han, R. Shi, X. Wu et al., Highly dispersed cobalt nanoparticles embedded in nitrogen-doped graphitized carbon for fast and durable potassium storage. Nano-Micro Lett. **13**, 21 (2020). 10.1007/s40820-020-00534-x 10.1007/s40820-020-00534-x PMC8187504 34138194

[29] S. Li, T. Xie, L. Ma, B. Li, D. Liu et al., Advanced bifunctional bionic neural network-like architecture constructed by multi-scale carbon nanotubes nanocomposites for enhanced microwave absorption. Compos. Part B Eng. **284**, 111714 (2024). 10.1016/j.compositesb.2024.111714

[30] Y. Wang, Y. Yang, M. Miao, X. Feng, Carbon nanotube arrays@cobalt hybrids derived from metal-organic framework ZIF-67 for enhanced electromagnetic wave absorption. Mater. Today Phys. 35, 101110 (2023). 10.1016/j.mtphys.2023.101110

[31] X. Lin, M. Han, Recent progress in soft electronics and robotics based on magnetic nanomaterials. Soft Sci. **3**, 14 (2023). 10.20517/ss.2023.05

[32] J. Xiao, B. Zhan, X. Qi, J. Ding, Y. Qu et al., Metal valence state modulation strategy to design core@shell hollow carbon Microspheres@MoSe_2_/MoO_*x*_ multicomponent composites for anti-corrosion and microwave absorption. Small e2311312 (2024). 10.1002/smll.202311312 10.1002/smll.202311312 38566552

[33] B. Fan, L. Xing, K. Yang, F. Zhou, Q. He et al., Synergistically enhanced heat conductivity-microwave absorption capabilities of g-C_3_N_4_@Fe@C hollow micro-polyhedra *via* interface and composition modulation. Chem. Eng. J. **451**, 138492 (2023). 10.1016/j.cej.2022.138492

[34] M. He, Hu. Jinwen, H. Yan, X. Zhong, Y. Zhang, P. Liu, J. Kong, Gu. Junwei, Shape anisotropic chain‐like CoNi/polydimethylsiloxane composite films with excellent low‐frequency microwave absorption and high thermal conductivity. Adv. Func. Mater. 2316691 2316691 (2024). 10.1002/adfm.202316691 10.1002/adma.202410186 39380425

[35] L. Ma, S. Li, M. Yan, N. Gao, F. Liu et al., Self-assembled hollow bowl-shaped metal-organic framework-derived electromagnetic wave absorbers with strong anti-microbiologically influenced corrosion performance. J. Alloys Compd. **949**, 169847 (2023). 10.1016/j.jallcom.2023.169847

[36] X. Li, W. You, C. Xu, L. Wang, L. Yang et al., 3D seed-germination-like MXene with *in situ* growing CNTs/Ni heterojunction for enhanced microwave absorption *via* polarization and magnetization. Nano-Micro Lett. **13**, 157 (2021). 10.1007/s40820-021-00680-w 10.1007/s40820-021-00680-w PMC8289940 34279760

[37] C. Xu, L. Wang, X. Li, X. Qian, Z. Wu et al., Hierarchical magnetic network constructed by CoFe nanoparticles suspended within “tubes on rods” matrix toward enhanced microwave absorption. Nano-Micro Lett. **13**, 47 (2021). 10.1007/s40820-020-00572-5 10.1007/s40820-020-00572-5 PMC8187526 34138216

[38] F. Wu, P. Hu, F. Hu, Z. Tian, J. Tang et al., Multifunctional MXene/C aerogels for enhanced microwave absorption and thermal insulation. Nano-Micro Lett. **15**, 194 (2023). 10.1007/s40820-023-01158-7 10.1007/s40820-023-01158-7 PMC10412520 37556089

[39] L. Gai, Y. Wang, P. Wan, S. Yu, Y. Chen et al., Compositional and hollow engineering of silicon carbide/carbon microspheres as high-performance microwave absorbing materials with good environmental tolerance. Nano-Micro Lett. **16**, 167 (2024). 10.1007/s40820-024-01369-6 10.1007/s40820-024-01369-6 PMC10987424 38564086

[40] G. Chen, H. Liang, J. Yun, L. Zhang, H. Wu et al., Ultrasonic field induces better crystallinity and abundant defects at grain boundaries to develop CuS electromagnetic wave absorber. Adv. Mater. **35**, e2305586 (2023). 10.1002/adma.202305586 37565983 10.1002/adma.202305586

[41] L. Xing, H. Cheng, Y. Li, Q. Chen, X. Liu, Simultaneous manipulation of constituent and structure toward MOFs-derived hollow Co_3_O_4_/Co/NC@MXene microspheres *via* pyrolysis strategy for high-performance microwave absorption. Chem. Eng. J. **487**, 150729 (2024). 10.1016/j.cej.2024.150729

[42] P. Liu, G. Zhang, H. Xu, S. Cheng, Y. Huang et al., Synergistic dielectric–magnetic enhancement *via* phase-evolution engineering and dynamic magnetic resonance. Adv. Funct. Mater. **33**, 2211298 (2023). 10.1002/adfm.202211298

[43] J.-C. Shu, Y.-L. Zhang, Y. Qin, M.-S. Cao, Oxidative molecular layer deposition tailoring eco-mimetic nanoarchitecture to manipulate electromagnetic attenuation and self-powered energy conversion. Nano-Micro Lett. **15**, 142 (2023). 10.1007/s40820-023-01112-7 10.1007/s40820-023-01112-7 PMC10232706 37258997

[44] G. Wang, C. Li, D. Estevez, P. Xu, M. Peng et al., Boosting interfacial polarization through heterointerface engineering in MXene/graphene intercalated-based microspheres for electromagnetic wave absorption. Nano-Micro Lett. **15**, 152 (2023). 10.1007/s40820-023-01123-4 10.1007/s40820-023-01123-4 PMC10247949 37286814

[45] M. Huang, B. Li, Y. Qian, L. Wang, H. Zhang et al., MOFs-derived strategy and ternary alloys regulation in flower-like magnetic-carbon microspheres with broadband electromagnetic wave absorption. Nano-Micro Lett. **16**, 245 (2024). 10.1007/s40820-024-01416-2 10.1007/s40820-024-01416-2 PMC11245463 38995472

[46] X. Liu, J. Zhou, Y. Xue, X. Lu, Structural engineering of hierarchical magnetic/carbon nanocomposites *via in situ* growth for high-efficient electromagnetic wave absorption. Nano-Micro Lett. **16**, 174 (2024). 10.1007/s40820-024-01396-3 10.1007/s40820-024-01396-3 PMC11018581 38619635

[47] Y. Wu, Y. Zhao, M. Zhou, S. Tan, R. Peymanfar et al., Ultrabroad microwave absorption ability and infrared stealth property of nano-micro CuS@rGO lightweight aerogels. Nano-Micro Lett. **14**, 171 (2022). 10.1007/s40820-022-00906-5 10.1007/s40820-022-00906-5 PMC9392679 35987861

[48] C. Yang, J. Zhang, M. Chang, J. Tan, M. Yuan et al., NIR-activatable heterostructured nanoadjuvant CoP/NiCoP executing lactate metabolism interventions for boosted photocatalytic hydrogen therapy and photoimmunotherapy. Adv. Mater. **36**, e2308774 (2024). 10.1002/adma.202308774 37917791 10.1002/adma.202308774

[49] Z. He, H. Yang, N.H. Wong, L. Ernawati, J. Sunarso et al., Construction of Cu_7_S_4_@CuCo_2_O_4_ yolk-shell microspheres composite and elucidation of its enhanced photocatalytic activity, mechanism, and pathway for carbamazepine degradation. Small **19**, e2207370 (2023). 10.1002/smll.202207370 36765447 10.1002/smll.202207370

[50] H. Qin, W. Zhang, S. Zhao, Z. Yao, Q. Zheng et al., Design of CoN/ZIS heterojunction with yolk-shell structure for impressive photocatalytic H_2_ evolution promoted by the photothermal effect. Chem. Eng. J. **489**, 151213 (2024). 10.1016/j.cej.2024.151213

[51] M. Wang, S. Zhu, Q. Huang, Y. Yan, M. Zhang et al., Near infrared light and tumor microenvironment multi-sensitive “Yolk-Shell MOF Nanoreactor” for synergistic treatment of cervical cancer. Chem. Eng. J. **498**, 155080 (2024). 10.1016/j.cej.2024.155080

[52] M. Li, Y. Sun, H. Lu, P. Zhu, R. Wang et al., Simultaneous improving luminescence intensity and stability of CsPbBr_3_: SCN–@Eu/Zr-uio-66-NH_2_ with tunable emissions from blue to green and applications in indoor photovoltaics. Nano Res. **17**, 6879–6887 (2024). 10.1007/s12274-024-6663-9

[53] Z. Xiang, Y. Shi, X. Zhu, L. Cai, Lu. Wei, Flexible and waterproof 2D/1D/0D construction of MXene-based nanocomposites for electromagnetic wave absorption, EMI shielding, and photothermal conversion. Nano-Micro Lett. **13**, 150 (2021). 10.1007/s40820-021-00673-9 10.1007/s40820-021-00673-9 PMC8233447 34170409

[54] L. Zhu, L. Tian, S. Jiang, L. Han, Y. Liang et al., Advances in photothermal regulation strategies: from efficient solar heating to daytime passive cooling. Chem. Soc. Rev. **52**, 7389–7460 (2023). 10.1039/D3CS00500C 37743823 10.1039/d3cs00500c

[55] JooHo Yun, Recent progress in thermal management for flexible/wearable devices. Soft Sci. **3**, 12 (2023). 10.20517/ss.2023.04

[56] W. Ma, P. He, Y. Zhou, C. Xie, Y. Chen et al., NiCo_2_O_4_/hollow mesoporous carbon nanosphere hybrids enabling super-hydrophobicity, thermal insulation, and highly efficient microwave absorption. Small **19**, e2305353 (2023). 10.1002/smll.202305353 37606896 10.1002/smll.202305353

[57] Z. Ma, J. He, S. Liu, X. Qie, M. Gan et al., Gradient layered MXene/Fe_3_O_4_@CNTs/TOCNF ultrathin nanocomposite paper exhibiting effective electromagnetic shielding and multifunctionality. Nano Res. **17**, 8233–8242 (2024). 10.1007/s12274-024-6824-x

[58] H. Liu, Y. Yang, N. Tian, C. You, Y. Yang, Foam-structured carbon materials and composites for electromagnetic interference shielding: design principles and structural evolution. Carbon **217**, 118608 (2024). 10.1016/j.carbon.2023.118608

[59] J. Yao, C. Kim, Q. Nian, W. Kang, Copper-graphene composite (CGC) conductors: synthesis, microstructure, and electrical performance. Small **20**, e2403241 (2024). 10.1002/smll.202403241 38984726 10.1002/smll.202403241

[60] S. Li, T. Xie, L. Ma, Z. Lei, N. Huang et al., Ni_3_Fe@N-doped carbon nanotubes 3D network induced by nanoconfined symmetry breaking for high-performance microwave absorption, corrosion protection, and pollutant purification. Carbon **213**, 118302 (2023). 10.1016/j.carbon.2023.118302

[61] P. Pan, Q. Liu, L. Hu, S. Liu, C. Wang et al., Dual-template induced interfacial assembly of yolk-shell magnetic mesoporous polydopamine vesicles with tunable cavity for enhanced photothermal antibacterial. Chem. Eng. J. **472**, 144972 (2023). 10.1016/j.cej.2023.144972

[62] N. Song, Y. Yu, Y. Zhang, Z. Wang, Z. Guo et al., Bioinspired hierarchical self-assembled nanozyme for efficient antibacterial treatment. Adv. Mater. **36**, e2210455 (2024). 10.1002/adma.202210455 36854170 10.1002/adma.202210455

[63] Y. Qiao, Y. Xu, X. Liu, Y. Zheng, B. Li et al., Microwave assisted antibacterial action of *Garcinia* nanoparticles on Gram-negative bacteria. Nat. Commun. **13**, 2461 (2022). 10.1038/s41467-022-30125-w 35513402 10.1038/s41467-022-30125-w PMC9072325

[64] Z. Lei, X. Zhang, L. Ma, B. Zheng, Y. Liu et al., Broadband microwave absorption and antibiosis effect of Cu@C@Fe_3_O_4_ nanocomposites. J. Alloys Compd. **1003**, 175497 (2024). 10.1016/j.jallcom.2024.175497

[65] W. Zhao, H. Zhou, W. Li, M. Chen, M. Zhou et al., An environment-tolerant ion-conducting double-network composite hydrogel for high-performance flexible electronic devices. Nano-Micro Lett. **16**, 99 (2024). 10.1007/s40820-023-01311-2 10.1007/s40820-023-01311-2 PMC10825113 38285132

[66] S. Chen, F. Huang, L. Mao, Z. Zhang, H. Lin et al., High Fe-loading single-atom catalyst boosts ROS production by density effect for efficient antibacterial therapy. Nano-Micro Lett. **17**, 32 (2025). 10.1007/s40820-024-01522-1 10.1007/s40820-024-01522-1 PMC11450126 39363132

[67] Y. Song, Q. Sun, J. Luo, Y. Kong, B. Pan et al., Cationic and anionic antimicrobial agents co-templated mesostructured silica nanocomposites with a spiky nanotopology and enhanced biofilm inhibition performance. Nano-Micro Lett. **14**, 83 (2022). 10.1007/s40820-022-00826-4 10.1007/s40820-022-00826-4 PMC8964905 35348927

[68] L. Jin, H. Liu, C. Wang, C. Mao, S. Wu et al., Interface/dipole polarized antibiotics-loaded Fe_3_O_4_/PB nanoparticles for non-invasive therapy of osteomyelitis under medical microwave irradiation. Adv. Mater. **36**, e2410917 (2024). 10.1002/adma.202410917 39344940 10.1002/adma.202410917

[69] R. Yu, H. Chen, J. He, Z. Zhang, J. Zhou et al., Engineering antimicrobial metal-phenolic network nanoparticles with high biocompatibility for wound healing. Adv. Mater. **36**, e2307680 (2024). 10.1002/adma.202307680 37997498 10.1002/adma.202307680

[70] L. Jin, S. Wu, C. Mao, C. Wang, S. Zhu et al., Rapid and effective treatment of chronic osteomyelitis by conductive network-like MoS_2_/CNTs through multiple reflection and scattering enhanced synergistic therapy. Bioact. Mater. **31**, 284–297 (2023). 10.1016/j.bioactmat.2023.08.005 37663620 10.1016/j.bioactmat.2023.08.005 PMC10469393

[71] C. Wang, C. Wang, S. Wu, Z. Cui, Y. Zheng et al., Microwave catalytic and thermal effects of Ti_3_C_2_T_x_/ZnO–PPy enhanced by interfacial polarization for rapid treatment of MRSA-induced osteomyelitis. Nano Today **58**, 102439 (2024). 10.1016/j.nantod.2024.102439

