# Supplementary material for: Yolk–Shell CoNi@N-Doped Carbon-CoNi@CNTs for Enhanced Microwave Absorption, Photothermal, Anti-Corrosion, and Antimicrobial Properties
Source: Nanomicro Lett. 2025 Feb 26;17:167. doi: 10.1007/s40820-024-01626-8 (PMC11865380; doi:10.1007/s40820-024-01626-8)
Supplement: Supplementary file 1 — Supplementary file1 (DOCX 19649 KB) [file 40820_2024_1626_MOESM1_ESM.docx]

Supporting Information for

**Yolk-Shell CoNi@N-Doped Carbon-CoNi@CNTs for Enhanced Microwave Absorption, Photothermal, Anti-Corrosion, and Antimicrobial Properties**

Qiqin Liang^1^, Mukun He^2^, Beibei Zhan^1^, Hua Guo^2^, Xiaosi Qi^1^,*, Yunpeng Qu^1^, Yali Zhang^1^, Wei Zhong^3^, Junwei Gu^2^,*

^1^ College of Physics, Guizhou Province Key Laboratory for Photoelectrics Technology and Application, Guizhou University, Guiyang City 550025, People’s Republic of China

^2^Shaanxi Key Laboratory of Macromolecular Science and Technology, School of Chemistry and Chemical Engineering, Northwestern Polytechnical University, Xi’an 710072, People’s Republic of China

^3^ National Laboratory of Solid State Microstructures and Jiangsu Provincial Laboratory for NanoTechnology, Nanjing University, Nanjing 210093, People’s Republic of China

*****Corresponding authors. E-mail: [xsqi@gzu.edu.cn](mailto:xsqi@gzu.edu.cn) (Xiaosi Qi), [gjw@nwpu.edu.cn](mailto:gjw@nwpu.edu.cn) (Junwei Gu)

**S1 Experimental Details**

**S1.1 Materials**

In the experiment, nickel sulfate hexahydrate (NiSO_4_·6H_2_O) and potassium hexacyanocobaltate (III) (K_3_[Co(CN)_6_]) were purchased from Shanghai Aladdin Biochemical Technology Co., Ltd. Trisodium citrate dihydrate (C_6_H_5_Na_3_O_7_·2H_2_O) was purchased from Jiangsu Qiangsheng Functional Chemistry Co., Ltd. Melamine was purchased from Shanghai Macklin Biochemical Technology Co., Ltd. All the used chemicals were analytical grade and used directly without further purification.

**S1.2 Fabrication of CoNi Prussian Blue Analogs (PBAs) Precursor**

In a typical experiment, 0.394 g of nickel sulfate hexahydrate and 0.662 g of trisodium citrate dihydrate are ultrasonically dispersed in 50 mL of deionized water. Meanwhile, 0.332 g of potassium hexacyanocobaltate (III) is also dissolved in 50 mL of deionized water. Subsequently, the obtained above-mentioned solutions are well mixed under a magnetic stirring and subsequently stood at room temperature for ca. 20 hours. After the repeated washed process and subsequently dried at 60 °C overnight, bimetallic CoNi PBAs can be acquired.

**S1.3 Synthesis of Yolk-shell CoNi@NC (N-doped carbon)-CoNi@Carbon Nanotubes (CNTs) Multicomponent Nanocomposites (MCNCs)**

Briefly, 0.25 g of CoNi PBAs and 1.25 g of melamine powder acting as carbon source are mixed and placed in a quartz boat, which is pushed into a quartz tube furnace (model BTF-1200C-S-SL, Anhui BEQ Equipment Technology CO., LTD). Afterward, the furnace is heated from room temperature to 300 °C and kept this temperature for 1 h under the atmosphere of Ar. Subsequently, as summarized in Table S1, the growth of CNTs is conducted at designed temperatures and times to produce CoNi@CN-CoNi@CNTs MCNCs, which are denoted as CoNi@CN-CoNi@CNTs-1, CoNi@CN-CoNi@CNTs-2, CoNi@CN-CoNi@CNTs-3 and CoNi@NC-CoNi@CNTs-4, respectively. Specially, the reference samples obtained from CoNi PBAs without melamine according to the pyrolysis conditions of CoNi@CN-CoNi@CNTs-1 were called CoNi@NC.

**Table S1** Summarized table for the main experimental conditions and obtained samples

| **Name of samples** | **Pyrolysis temperature (℃)** | | **Pyrolysis time (h)** |
| --- | --- | --- | --- |
| CoNi@NC-CoNi@CNTs-1 | 660 | 2 | |
| CoNi@NC- CoNi@CNTs-2 | 760 | 2 | |
| CoNi@NC- CoNi@CNTs-3 | 860 | 2 | |
| CoNi@NC- CoNi@CNTs-4 | 660 | 4 | |

**S1.4 Materials Characterization**

To investigate the phases, microstructures and chemical compositions, the as-prepared samples were characterized by X-ray powder diffractometry (XRD) (model Smart Lab, Rigaku) using Cu-Kα radiation, scanning electron microscopy (SEM) (model Hitachi Regulus 8100) and transmission electron microscopy (TEM) (model Talos F2100X, Thermo Fisher Scientific), respectively. Raman spectroscopy (model DXR, Thermo Fisher Scientific) and X-ray photoelectron spectroscopy (XPS, Escalab Xi^+^). The water contact angles (WCA) were measured using 5 μL DI droplet at RT, which were conducted on a contact angle meter, combined with a high-speed camera. The sample was mixed with polyvinylidene difluoride (PVDF) into a battery containing 22 wt% sample, and the AC impedance was measured through a chemical workstation. For the measurement of their EMWAPs, the as-prepared samples (22 wt%) were firstly mixed with paraffin and subsequently pressed into a toroidal shape (inner diameter: 3.0 mm, outer diameter: 7.0 mm), and then their EM parameters were measured over a vector network analyzer (R&S ZNB-40) in the 2-18 GHz frequency range using the coaxial-line method. According to the transmission line theory, the values of reflection loss (RL) value and attenuation constant ($\alpha$) could be obtained on basis of the measured EM parameters and the following equations [3, 7]:

$RL=20\log\left|\frac{Z_{in}-Z_{0}}{Z_{in}+Z_{0}}\right|$ (S1)

$Z_{in}=Z_{0}\sqrt{\frac{\mu}{\varepsilon}}\tanh\left(j\frac{2\pi fd\sqrt{\mu\varepsilon}}{c}\right)$ (S2)

$Z=\frac{Z_{\text{in}}}{Z_{0}}$ (S3)

$\alpha=\frac{\sqrt{2}\pi f}{c}\sqrt{\left(\mu''\varepsilon''-\mu'\varepsilon'\right)+\sqrt{\left(\mu''\varepsilon''-\mu'\varepsilon'\right)^{2}+\left(\mu''\varepsilon''+\mu'\varepsilon'\right)^{2}}}$ (S4)

where $Z_{in}$—input impedance, $Z_{0}$—impedance of the free space,$\varepsilon=\varepsilon'-\text{j}\varepsilon''$—complex permittivity, $\mu=\mu'-\text{j}\mu''$—complex permeability, f—frequency, d—thickness and c—velocity of light, respectively.

The radar cross-section (RCS) values of CoNi@NC-CoNi@CNTs MCNCs were simulated using CST Studio Suite 2020. A smaller RCS value indicates better electromagnetic (EM) wave stealth performance [8]. The simulation employed a linear polarization wave as the excitation source and monitored the frequency at 11.2 GHz.

**S1.5 In Vitro Antibacterial Test**

0.1 mg CoNi@NC-CoNi@CNTs sample was mixed with 1 mL bacterial solution (0.1×10^6^ CFU/mL, CFU= colony-forming unit), respectively. The suspension was ultrasonicated for 10 min, and incubated in a thermostatic oscillator at 37 °C for 24 h. Then, the bacterial suspension was continuously diluted with sterile PBS solution. 100 μL bacterial suspension was uniformly spread on the Luria-Bertani (LB) solid medium and incubated at 37 °C for 18 h. Finally, the number of colonies was counted and the antibacterial rate was calculated. A blank control group (CK) was set up in the experiment. The strains used Escherichia coli (gram-negative bacteria) in the experiment.

**S1.6 In Anti-corrosion Test**

The samples were immersed in 3.5 wt% NaCl solution and measured for their open circuit potential (OCP), AC impedance, and Tafel plots via a chemical workstation.

**S2 Supplementary Figures and Tables**


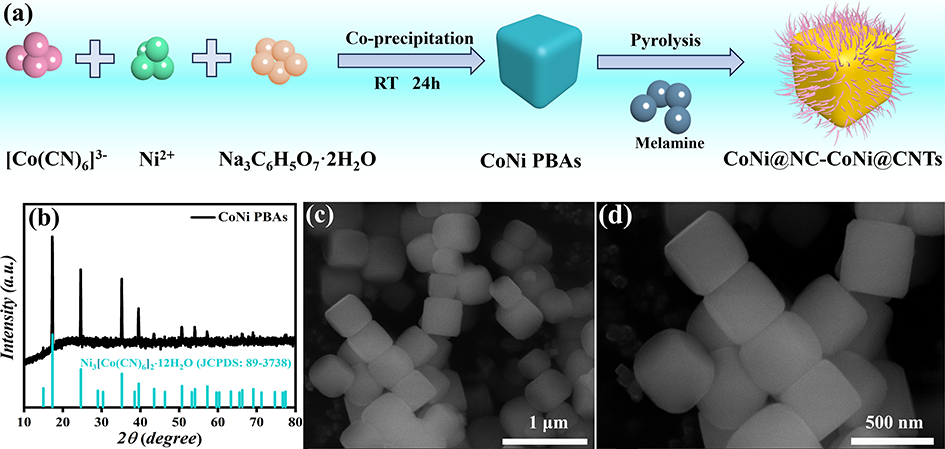


**Fig. S1 a** Schematic illustration of the synthesis of CoNi@NC-CoNi@CNTs, **b** XRD pattern, **c** and **d** SEM images of CoNi PBAs

**
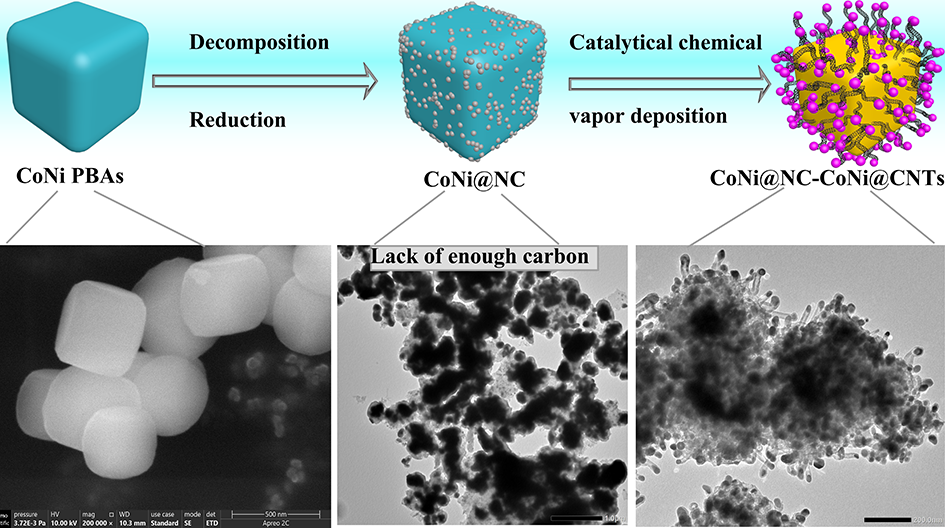
**

**Fig. S2** Illustration for growth mechanism of CoNi@NC-CoNi@CNTs derived from CoNi PBAs


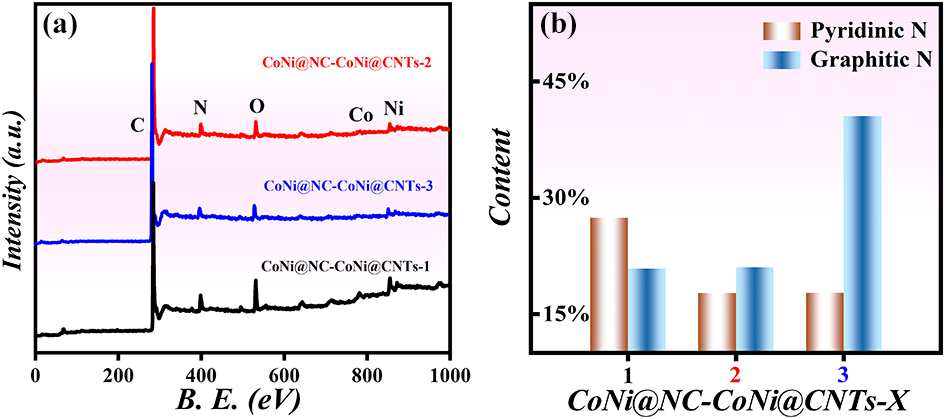


**Fig. S3 a** Survey scan XPS spectra and **b** the contents of various N for CoNi@NC-CoNi@CNTs-1, CoNi@NC-CoNi@CNTs-2 and CoNi@NC-CoNi@CNTs-3, respectively


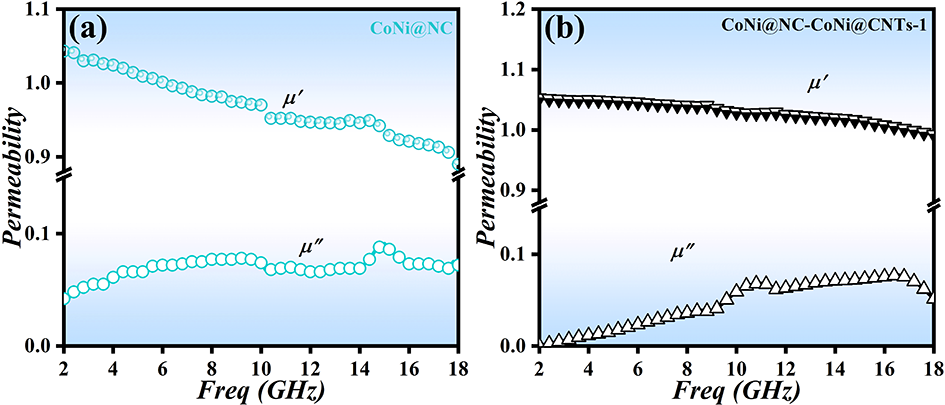


**Fig. S4** Complex permeability of **a** CoNi@NC and **b** CoNi@NC-CoNi@CNTs-1


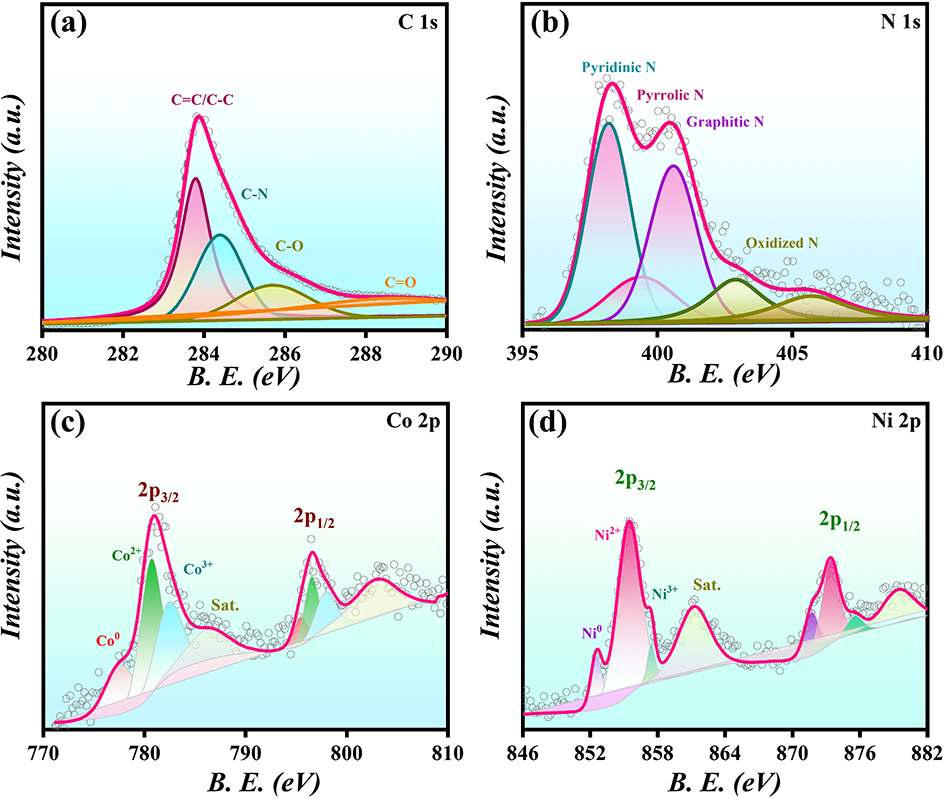


**Fig. S5** High resolution XPS spectra of C 1s, N1s, Co 2p and Ni 2p for CoNi@NC-CoNi@CNTs-4


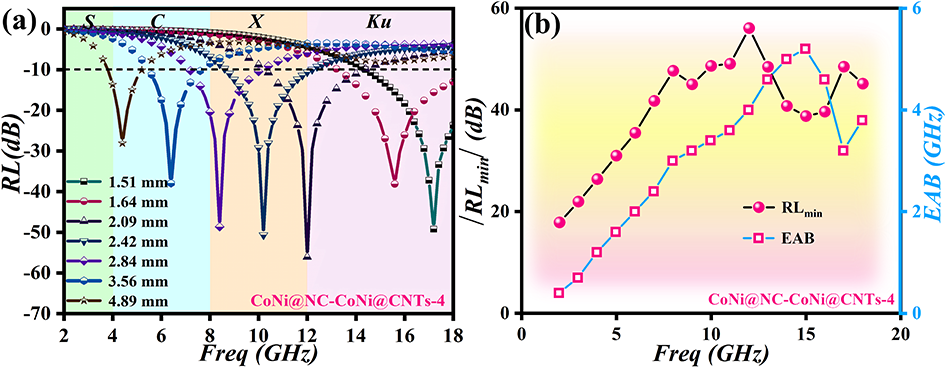


**Fig. S6** **a** RL values, **b** RL_min_ and EAB values for CoNi@NC-CoNi@CNTs-4


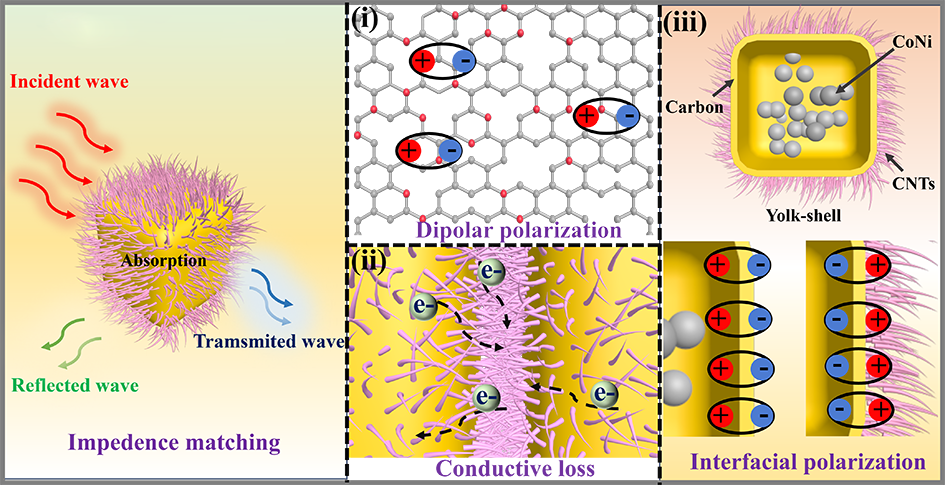


**Fig. S7** EM wave attenuation avenues of [CoNi@NC-CoNi@CNTs](mailto:CoNi@NC-CoNi@CNTs.--)


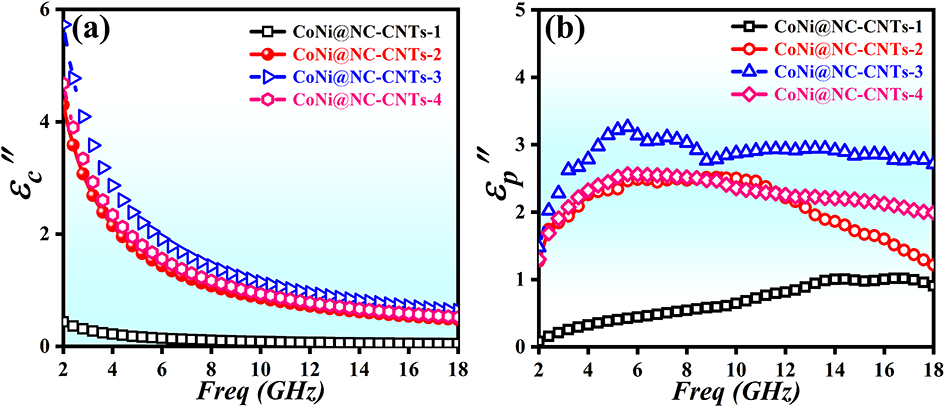


**Fig. S8** **a** and **b** $\varepsilon''_{c}$ and $\varepsilon''_{p}$ values of CoNi@NC-CoNi@CNTs


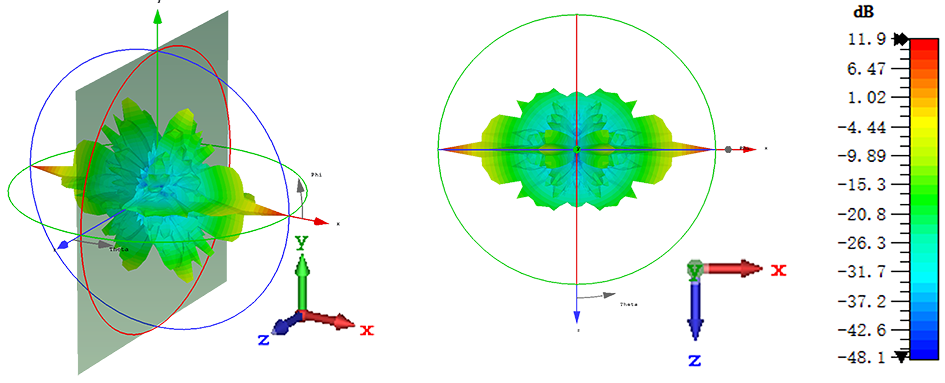


**Fig. S9** Simulation results to pure PEC


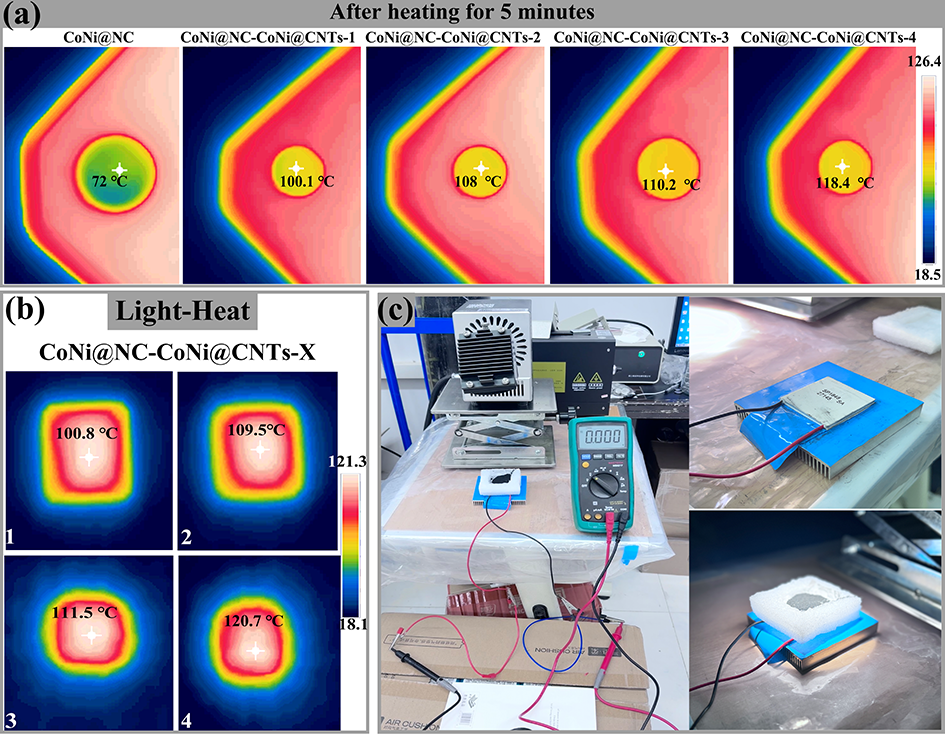


**Fig. S10 a** Infrared thermography photos, **b** infrared thermography photos after 10 min of light exposure **c** self-built solar-thermoelectric generator for CoNi@NC-CoNi@CNTs MCNCs


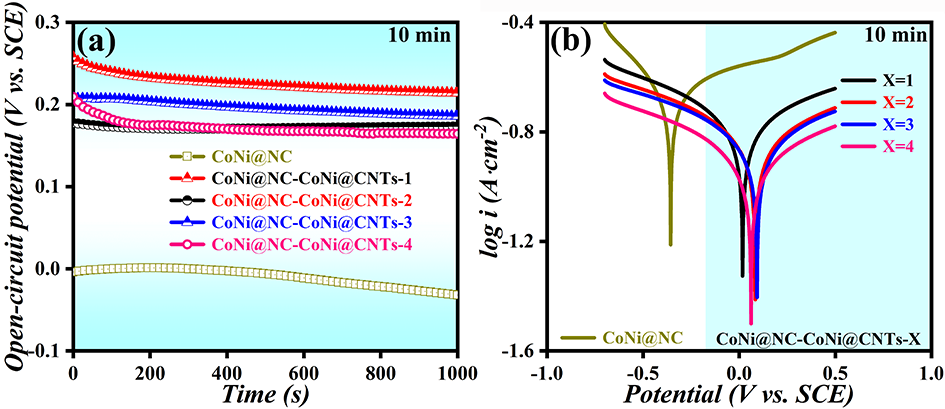


**Fig. S11 a** OCP plots and **b** Tafel plots of CoNi@NC and CoNi@NC-CoNi@CNTs.


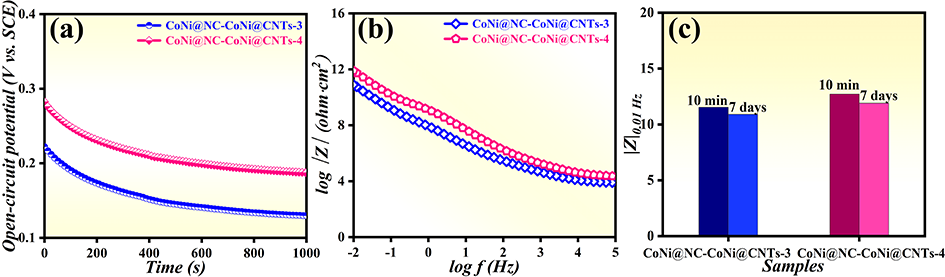


**Fig. S12 a** OCP plot, **b** impedance modulus curves and **c** |Z|_0.01_ _Hz_ values of CoNi@NC-CoNi@CNTs-3 and CoNi@NC-CoNi@CNTs-4


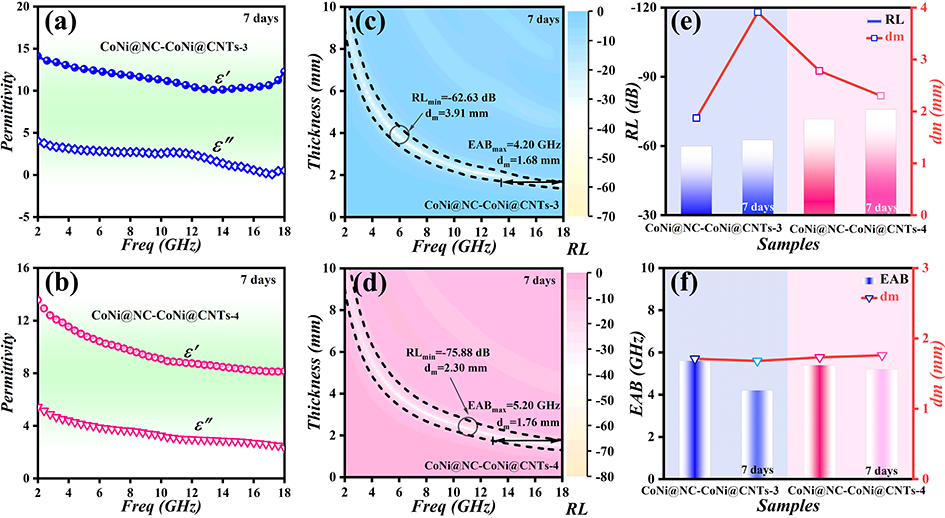


**Fig. S13 a**, **b** Complex permittivity, **c**, **d** RL color maps, **e** RL values and **f** EAB values of CoNi@NC-CoNi@CNTs MCNCs immersed in 3.5 wt% NaCl solution for 7 days


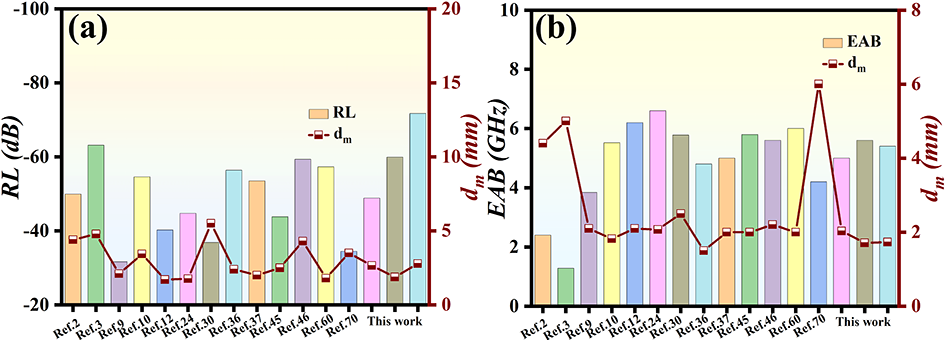


**Fig. S14** Summarized RL_min_ and EAB, and d_m_ values between representative MCNCs and our designed CoNi@NC-CoNi@CNTs MCNCs

**Table S2** Comparison Antibacterial performances with several representative works

| **Materials** | **Concentration** | **Antibacterial ratio** | **Refs** |
| --- | --- | --- | --- |
| Co/C | compared with CK | 5.63% | [35] |
| YS-MMP@Cur | 1 mg/mL | 99.2% | [61] |
| NiCo_2_O_4_ nanozyme | 1 mg/ml | 99.62% | [62] |
| MRSA | 0.21 mg/ml | 30.56 % | [63] |
| Cu@C@Fe_3_O_4_-450 | 0.1 mg/mL | 90% | [64] |
| h^3^-FNC | 0.22 mM | 70% | [66] |
| BAC/NaSal | 4 ug/mL (BAC) | 92.41% | [67] |
| PLGA/Fe_3_O_4_/PB/Gent | 1 mg/mL | 99.9% | [68] |
| am-MPN | 5 uM | 68% | [69] |
| MoS_2_/CNTs | 1 mg/ml | 86.37% | [70] |
| Ti_3_C_2_T_x_/ZnO-PPy | 0.03 mg/mL | 99.11 % | [71] |
| [CoNi@NC-CoNi@CNTs-1](mailto:CoNi@NC-CoNi@CNTs-1) | 0.1 mg/mL | 70.96% | This work |
| [CoNi@NC-CoNi@CNTs-2](mailto:CoNi@NC-CoNi@CNTs-2) |  | 78.81% |  |
| [CoNi@NC-CoNi@CNTs-3](mailto:CoNi@NC-CoNi@CNTs-3) |  | 81.84% |  |
| [CoNi@NC-CoNi@CNTs-4](mailto:CoNi@NC-CoNi@CNTs-4) |  | 84.03% |  |
